# Microenvironmental Regulation of Macrophage Transcriptomic and Metabolomic Profiles in Pulmonary Hypertension

**DOI:** 10.3389/fimmu.2021.640718

**Published:** 2021-03-31

**Authors:** Min Li, Suzette Riddle, Sushil Kumar, Joanna Poczobutt, B. Alexandre McKeon, Maria G. Frid, Maureen Ostaff, Julie A. Reisz, Travis Nemkov, Mehdi A. Fini, Aya Laux, Cheng-Jun Hu, Karim C. El Kasmi, Angelo D’Alessandro, R. Dale Brown, Hui Zhang, Kurt R. Stenmark

**Affiliations:** ^1^ Cardiovascular Pulmonary Research Laboratories, Departments of Pediatrics and Medicine, University of Colorado Anschutz Medical Campus, Aurora, CO, United States; ^2^ Department of Biochemistry and Molecular Genetics, University of Colorado Anschutz Medical Campus, Aurora, CO, United States; ^3^ Department of Craniofacial Biology, School of Dental Medicine, University of Colorado Anschutz Medical Campus, Aurora, CO, United States

**Keywords:** adventitial fibroblast, macrophage, transcriptomic, metabolomics, mitochondria, C-terminal binding protein-C, pulmonary hypertension

## Abstract

The recruitment and subsequent polarization of inflammatory monocytes/macrophages in the perivascular regions of pulmonary arteries is a key feature of pulmonary hypertension (PH). However, the mechanisms driving macrophage polarization within the adventitial microenvironment during PH progression remain unclear. We previously established that reciprocal interactions between fibroblasts and macrophages are essential in driving the activated phenotype of both cell types although the signals involved in these interactions remain undefined. We sought to test the hypothesis that adventitial fibroblasts produce a complex array of metabolites and proteins that coordinately direct metabolomic and transcriptomic re-programming of naïve macrophages to recapitulate the pathophysiologic phenotype observed in PH. Media conditioned by pulmonary artery adventitial fibroblasts isolated from pulmonary hypertensive (PH-CM) or age-matched control (CO-CM) calves were used to activate bone marrow derived macrophages. RNA-Seq and mass spectrometry-based metabolomics analyses were performed. Fibroblast conditioned medium from patients with idiopathic pulmonary arterial hypertension or controls were used to validate transcriptional findings. The microenvironment was targeted *in vitro* using a fibroblast-macrophage co-culture system and *in vivo* in a mouse model of hypoxia-induced PH. Both CO-CM and PH-CM actively, yet distinctly regulated macrophage transcriptomic and metabolomic profiles. Network integration revealed coordinated rewiring of pro-inflammatory and pro-remodeling gene regulation in concert with altered mitochondrial and intermediary metabolism in response to PH-CM. Pro-inflammation and metabolism are key regulators of macrophage phenotype *in vitro*, and are closely related to *in vivo* flow sorted lung interstitial/perivascular macrophages from hypoxic mice. Metabolic changes are accompanied by increased free NADH levels and increased expression of a metabolic sensor and transcriptional co-repressor, C-terminal binding protein 1 (CtBP1), a mechanism shared with adventitial PH-fibroblasts. Targeting the microenvironment created by both cell types with the CtBP1 inhibitor MTOB, inhibited macrophage pro-inflammatory and metabolic re-programming both *in vitro* and *in vivo*. In conclusion, coordinated transcriptional and metabolic reprogramming is a critical mechanism regulating macrophage polarization in response to the complex adventitial microenvironment in PH. Targeting the adventitial microenvironment can return activated macrophages toward quiescence and attenuate pathological remodeling that drives PH progression.

## Introduction

Pulmonary Hypertension (PH) is a progressive and devastating disease characterized by both vascular inflammation and structural remodeling (1–7). Current therapies primarily function as vasodilators and provide symptomatic relief but do not halt progressive remodeling (8). Myeloid leukocytes, especially macrophages, are thought to be primary effectors of inflammation and remodeling in the diseased PH vessel wall (9, 10). Indeed, macrophages are key players in maintaining tissue homeostasis under normal conditions. In pathologic conditions, recruitment of circulating monocyte/macrophages to the pulmonary arterial wall, especially the adventitial compartment, is a prerequisite for vascular remodeling and development of PH (1, 4, 10–17).

A critical observation in humans with PH as well as in various small and large animal models of PH is that macrophage accumulation is largely restricted to the adventitial/perivascular compartment of the vessel wall, implying an important function for the adventitia and adventitial macrophages in the vascular remodeling process in PH pathophysiology (5, 9, 18). It is recognized that the composition of the local tissue microenvironment (i.e., the relative concentrations of metabolites, cytokines, growth factors, oxygen, etc.), which include signals from other cell types in the proximate environment, are critical determinants of macrophage metabolism and function (19–23). Changes in cellular metabolism in macrophages act as critical regulators of macrophage functional response, and are important primary indicators of altered tissue homeostasis (24–27). Macrophages read out microenvironmental signals by undergoing metabolic and transcriptional reprogramming (23, 28, 29). Furthermore, the cellular metabolism of macrophages may directly affect the microenvironment and other cells contained therein through generation and/or depletion of metabolic signals and/or cytokines. Therefore, it is logical to believe that the functional phenotype of resident or recruited macrophages changes dynamically in response to local environmental and metabolic cues. These considerations are likely to be especially important in diseases of chronic and progressive inflammatory-fibrotic remodeling such as PH. It is generally understood that macrophages polarize from quiescence in normal tissue to activated state(s) in response to injurious stimuli. However, little is known of the specific metabolic and transcriptomic re-programming of pulmonary artery adventitial macrophages under homeostatic or pathologic conditions.

A clearly important aspect of homeostatic tissue function with regard to macrophages is their interaction and/or communication with other cell types. In the case of the normal and abnormal pulmonary artery adventitia where macrophages localize, the principal resident stromal cell is the fibroblast. Previous reports from our laboratory have documented that adventitial fibroblasts from animals and humans with PH (PH-Fibs) exhibit an epigenetically altered phenotype characterized by pro-inflammation, anti-apoptosis and high proliferation. These phenotypes are maintained in culture even under normoxic conditions (30). Further, the presence of a miR124-PTBP1 (polypyrimidine tract binding protein 1)-PKM (pyruvate kinase muscle) axis (31) in both bovine and human PH-Fibs results in active metabolic reprogramming toward to an aerobic glycolytic state characterized by increased free NADH (nicotinamide adenine dinucleotide) levels and activation of the transcriptional co-repressor C-terminal binding protein 1 (CtBP1) (32). These pathways collectively integrate fibroblast metabolism with the expression of gene products (Il-1β, IL-6, CCL2/MCP1, and CXCL12/SDF1) involved in PH pathogenesis.

PH-Fibroblasts have the ability to polarize naïve macrophages toward a distinct activated phenotype (19). Importantly, explanted adventitial tissue from animals with PH was also capable of activating macrophages to this phenotype (19). Additionally, these studies revealed that this macrophage activated phenotype is, in part, mediated by PH-Fibroblast-derived paracrine IL-6, a canonical activator of STAT-3 signaling in macrophages (19, 33). This role for IL-6 is interesting and consistent with the fact that it has been widely reported to be an important cytokine in the pathogenesis of PH in patients and animal models. However, IL-6 production alone (by one cell type or many) may not be solely responsible for PH disease outcome (34). A recent study has demonstrated that targeting IL-6 fails to abrogate the pulmonary hypertensive response (35). An alternative idea has been proposed, namely, that a positive feedback loop or interactive communication between activated fibroblasts and macrophages contributes to the chronic, perpetuated inflammation observed in PH. Supporting evidence for this concept comes from studies showing that an activated phenotype of tumor-associated macrophages, characterized by increased expression of arginase-1, HIF-1, and VEGF, derives from paracrine signals generated by cancer cells (28, 36).

We propose to examine the possibility that macrophages undergo distinct transcriptomic and metabolomic programing in response to and in coordination with signals generated from fibroblasts in both control and pulmonary hypertensive adventitial microenvironments to either maintain homeostasis or adapt to disease.

Collectively, our studies provide the first comprehensive analysis of the macrophage transcriptomic and metabolic phenotype that is generated in response to the complex signals derived from fibroblasts in the adventitia to which monocyte/macrophages are recruited in PH. They further provide evidence that targeting the fibroblast-macrophage interaction at the level of abnormal metabolism *via* inhibiting the metabolic sensor CtBP1 within this microenvironment could be useful as a method for abrogating persistent inflammation in PH.

## Materials and Methods

Materials and methods are summarized here; further detailed procedures are provided in the **Supplementary Material**.

### Animals

Neonatal male Holstein calves were exposed to hypobaric hypoxia for 2 weeks, while age-matched controls were kept at ambient altitude.

All mice were C57bl6/J background. Wildtype and *Hif1α* ^fl/fl^;LysMcre were purchased from Jackson Laboratories (Bar Harbor, Maine, USA). Mice were kept at simulated sea level altitude for one week after arrival. Mice were then randomly separated into three groups: 1) sea level normoxia, 2) hypoxia + PBS, and 3) hypoxia + 4-methylthio-2-oxobutanoic acid (MTOB). Hypoxic groups were placed in hypobaric hypoxic chambers for 4 days, corresponding to the peak of macrophage recruitment and inflammation observed previously.

### Cell Culture

Adventitial fibroblasts from distal pulmonary arteries of calves were isolated by explant culture. Human pulmonary artery fibroblasts were derived from patients with idiopathic PAH or from control donors undergoing lobectomy or pneumonectomy. All cells were cultured under ambient (Denver) normoxic conditions. Conditioned media (CM) was collected after 1 day of growth when the cells were greater than 85% confluent at the time of collection. Immediately prior to use, CM was supplemented with 10%FBS,enotoxin levels < 0.3 EU. Macrophages were treated with media conditioned by CO-Fibs (CO-CM) or PH-Fibs (PH-CM) or left untreated (UNX) for 18 hours for RNA isolation. For co-culture, PH-Fibs were either left untreated or pre-treated with CtBP1 inhibitor, MTOB. Prior to exposing BMDM to pre-treated PH-Fibs culture, the media was supplement with 1/3 final volume of fresh media, with or without drug. The BMDMs were exposed to PH-Fibs through a Transwell system for 18hrs prior to harvest.

### Mouse Lung Interstitial/Perivascular Macrophage Preparation and Flow Cytometry

Interstitial/perivascular macrophages were isolated by flow cytometry from total lung digests using the following gating strategy: Debris, doublets and dead cells were excluded, intravascular cells were removed by sorting against pre-injected intravenous CD45 antibody, macrophages were identified by sorting on CD64, and then interstitial/perivascular macrophages were separated from alveolar macrophages by high-CD11b/low-CD11c vs. high-CD11c/lowCD11b, respectively.

### RNA Sequencing (RNA-Seq) and Bioinformatics Analysis

RNA-Seq library preparation and sequencing were conducted using a NuGen universal plus mRNA-Seq kit. Sequencing was performed on Illumina NovaSEQ6000 system, using the paired-read 2x150 cycle option. RNA-Seq reads were generated using Illumina NovaSEQ6000 analysis pipeline. In this study we also included our previously published interstitial macrophage raw data (13), archived at https://www.ebi.ac.uk/ena/data/view/PRJNA345360. The read quality of all the samples were checked using FastQC v0.11.5. Adapter trimming, quality control, and base correction were performed by AfterQC. EdgeR in R was used for data normalization and differential expression analysis of RNA-Seq expression profiles with biological replication. The Benjamini-Hochberg procedure was used to control the false discovery rate (FDR), and a cut-off criterion of FDR ≤ 0.05 was applied to identify differentially expressed genes. Individual gene expression was calculated as ‘reads per kilobase per million mapped reads’ (RPKM).

### Quantitative RT-PCR

Quantitative Real-Time PCR was performed using either TaqMan probes or SybrGreen primers (**Supplemental Table 1**).

### Ultra-High Pressure Liquid Chromatography-Mass Spectrometry-Based Metabolomics Analyses

BMDMs were treated with CO-CM, PH-CM, or left untreated for 18hrs, and then pelleted and lysed at a concentration of 3 x 10^6^ cells/ml in ice-cold buffer for analysis. The analytical platform employs a 5 minute C18 gradient on a Vanquish UHPLC system coupled to a Q Exactive mass spectrometer in positive and negative ion modes (separate runs).

### Integration of Transcriptional and Metabolic Data

RNA-Seq and metabolomics data of BMDMs treated with CO-CM or PH-CM were analyzed together using MetaboAnalyst (https://www.metaboanalyst.ca/). All differentially expressed genes and metabolites after treatment with conditioned media were uploaded to generate overall interaction networks. For specific metabolite modules, all metabolites detected in the specific pathway were uploaded regardless of their differential expression and analyzed with all differentially expressed genes to create a complete subnetwork.

### Measurement of Glycolysis and Oxygen Consumption Rate by Seahorse Analysis

The Seahorse Bioscience XF96 Extracellular Flux analyzer (Agilent, North Billerica, MA, USA) was used to evaluate the mitochondrial respiration of BMDMs by measurement of oxygen consumption rate (OCR) and extracellular acidification rate (ECAR). These studies were supported in part by the Molecular and Cellular Analytical core in the Colorado Nutrition Obesity Research Center (P30 DK48520).

### Fluorescence Lifetime Imaging Microscopy (FLIM)

BMDMs were treated with PH-CM for 18hrs prior to measurement of intracellular NADH levels by fluorescence lifetime imaging microscopy (FLIM). FLIM of two-photon excited NADH was performed at the UCD Advanced Light Microscopy Core to directly quantitate NADH level in cells.

### Assessment of NADH/NAD+ Ratio

NADH/NAD+ ratio was determined using modified methods of Williamson (37), which is based on the enzymatic cycling reaction (Pyruvate + NADH + H+ ↔ Lactate + NAD+) and was performed as previously described (38).

### Immunostaining Staining

5 μm lung tissue sections were stained with primary antibodies against mouse, bovine and human CtBP1, macrophage marker CD68 and tenascin C (TNC). Immunolabeled sections were examined under a Zeiss fluorescent microscope, and images were acquired using AxioVision digital imaging system. Human pulmonary artery specimens from rejected normal donors (n=3) and patients with pulmonary (arterial) hypertension (PH/PAH) (n=5) (see **Supplemental Table 2** for detailed description of clinical characteristics, demographics and pathological diagnoses) were obtained from Pittsburg University Tissue Bank. Samples were de-identified and were used secondarily after other collection purposes. Pittsburg University Tissue Bank has obtained permission to study the tissues obtained.

### Statistical Analysis

Prism software version 7 (GraphPad, Software, Inc, www.graphpad.com) was used for t-test, one-way, or two-way ANOVA followed by Bonferroni post-test analysis for multiple comparisons. Values were expressed as mean ± SEM. For basic comparisons of two Gaussian distributed sample sets, we used Student’s unpaired, two-tail t-tests. When comparing multiple groups, the respective ANOVA (one way when comparing one characteristic, or two ways if two dependent variables were involved) was performed. P-values were subject to multiple testing adjustments using Bonferroni correction. Differences with *p* values less than 0.05 were considered statistically significant.

## Results

### Conditioned Media From Control and Pulmonary Hypertensive Adventitial Fibroblasts Elicit Distinct Macrophage Phenotypes and Transcriptional Profiles

**Figure 1:**
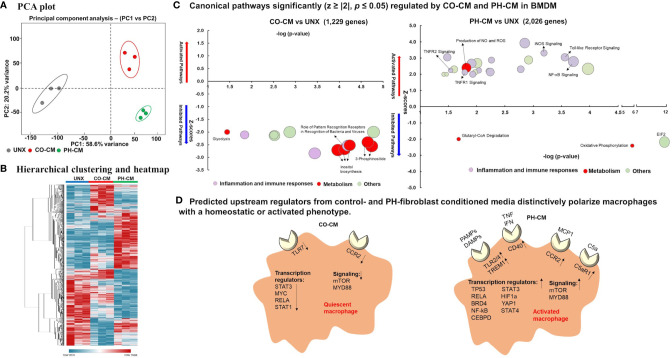
Control and pulmonary hypertensive adventitial fibroblast conditioned media actively, yet differently, regulate macrophage gene profile. Naïve bone marrow derived macrophages (BMDMs) were treated with media conditioned by pulmonary artery adventitial fibroblasts isolated from 2-week old hypoxia induced pulmonary hypertensive (PH-CM) or age-matched control calves (CO-CM), or left untreated (UNX). 12,191 genes met quality control standards and with count greater than 1. **(A)** Principal component analysis (PCA) showing overall separation of CO-CM and PH-CM treated BMDMs from untreated BMDMs. Each dot represents RNA-Seq data from biologic triplicates. **(B)** Heat-map showing unsupervised hierarchical clustering of genes from RNA-seq that are uniquely regulated by CO-CM or PH-CM and analyzed by Ingenuity Pathway Analysis (IPA). **(C)** The significantly regulated canonical pathways of BMDMs by these genes demonstrated that CO-CM (left) and PH-CM (right) have very different effects on regulating BMDM phenotypes. The pathways were selected with p ≤ 0.05, absolute Z ≥ 2. The size of the bubble indicates the number of genes in that pathway. **(D)** IPA predicted upstream regulators in CO-CM (left) and PH-CM (right) treated BMDMs (p ≤ 0.05, absolute Z ≥ 2). Upstream Regulators are transcripts, proteins, or metabolites that are predicted based on the downstream targets identified by RNA-seq. The upstream regulators from CO-CM included: 1) decreased receptors of CCR2 and TLR7, 2) decreased transcription regulators, and 3) decreased signaling pathways. In contrast, the upstream regulators from PH-CM included: 1) increased receptors for pathogen-associated molecular pattern molecules (PAMPs) and damage-associated molecular patterns (DAMPs) (Toll-like receptors, TREM1) and other receptors (CD40, CCR2, C5aR1), 2) increased transcription regulators, and 3) increased signaling pathways.

Previous work from our group demonstrated that PH-Fibs exhibit a persistently activated phenotype with increased production of cytokines, growth factors and the aerobic glycolysis end product, lactate (30, 32). We also established that an IL6/STAT3/HIF signaling pathway is involved in macrophage activation by PH-Fibs (19). We thus evaluated the effects of a combination of IL6+Lactate on macrophage gene expression, compared with the effects of whole PH-Fib conditioned medium. We found that even though IL6, lactate, or IL6+lactate can stimulate *IL1b*, *Vegf, Arg1, Asl* and *Ass1* gene expression in BMDM, the level of the induction is far less than that of complete PH-CM (**Supplemental Figure 1**). This result motivated us to comprehensively evaluate how macrophages and recruited monocytes respond to the complex adventitial microenvironmental signals created in part by adventitial fibroblasts in physiologic or pathologic settings. We treated naïve mouse BMDMs with cell culture media conditioned by adventitial fibroblasts isolated from either pulmonary hypertensive (PH-CM) or age-matched control neonatal calves (CO-CM), and performed RNA-Seq analysis. After data processing as described in the methods and shown in **Supplemental Figure 2**, the results demonstrated that both CO-CM and PH-CM had significant, yet vastly different, effects on gene expression in BMDMs (**Figure 1**). Principal component analysis (PCA, **Figure 1A**) of all 12,191 genes showed overall separation of CO-CM and PH-CM treated BMDMs from untreated BMDMs. Each dot represents RNA-Seq data from biological triplicates.

The top 30 genes up-regulated by PH-CM and the top 30 genes down-regulated by CO-CM compared to untreated BMDMs are shown in **Table 1**. PH-CM remarkably stimulated the expression of genes involved in inflammation [*Ackr3/Cxcr7* (a receptor for SDF1) and Il1b], metabolism [*Arg2, Slc36a2, and Slc7a11* (cysteine/glutamate transporter)], and cell recruitment/adhesion as well as vascular/extra cellular matrix (ECM) remodeling (*Thbs1, Itga1*/Integrin alpha-1, *Vcan*/Versican and *Pcam1*). The top inhibited genes by CO-CM involve inflammation (*Ccl12, Ccl7*, and *Ccl2*/*Mcp1*), cholesterol metabolism (*Ch25d*), and cell cycle (*Ccnd1, Ccne2*, and *Mybl2*) supporting the findings in IPA that CO-CM inhibited monocyte recruitment, growth and activation (**Figure 1C**). We compared the genes induced by PH-conditioned media to genes previously reported to characterize M1 vs. M2 macrophage phenotypes. We identified a distinct phenotype in PH-CM stimulated macrophages which exhibited a multi-dimentional phenotype with increased expression of both M1 and M2 marker genes (**Supplemental Figure 3**).

**Table 1:** Top 30 genes up-regulated by PH-CM and down-regulated by CO-CM in BMDMs.

PH-CM/UNX			CO-CM/UNX		
Genes	FC	p-value	Genes	FC	p-value
Rab44	474.53	9.17E-97	Ccl12: monocyte chemotactic Protein 5	-274.73	1.49E-168
Gm16194	168.74	1.49E-42	Ccl12: monocyte chemotactic Protein 3	-78.23	1.83E-245
Thbs1	113.72	5.18E-127	Ccl2/MCP1	-49.31	2.98E-117
Askr3/Cxcr7	108.99	6.83E-29	Cd300e	-39.47	1.40E-109
Cd2	97.47	5.81E-63	Ambp	-38.89	1.53E.28
Arg2	74.39	1.12E-50	Ctnnd2	36.40	3.51E-169
Bex1	71.88	1.02E-12	Dmpk	-35.92	5.47E-256
II1b	60.55	5.00E-28	Gm26902	-32.28	7.16E-12
Ms4a4a	59.36	0.00E+00	Lrrc4	-30.09	6.18E-32
Mcemp1	51.59	2.19E-75	Lipg	-28.94	5.63E-18
SIpi	49.01	1.48E-42	Ch25h	-18.39	1.65E-16
Mefv	45.36	4.89E-30	Ccnd1	-18.26	1.70E-92
Itga1/Integrin alpha-1	44.59	6.01E-196	Dmwd	-17.00	7.14E-136
Adgre4	39.04	1.16E-39	Bcl6b	-16.26	2.42E-23
Vcan	38.73	2.11E-273	Slco4a1	-15.61	3.50E-71
Gm16587	37.74	3.75E-37	St3gaI6	-15.10	1.69E-64
Myom1	35.80	5.40E-85	Cass4	-14.63	1.18E-38
Pecam1	35.79	2.61E-31	Ung	-13.23	7.94E-86
Igfbp5	23.06	2.26E-43	Id1	-12.56	2.84E-47
Nt5e	22.81	3.42E-47	Nid2	-12.14	4.65E-95
SIc7a11 Cystine/Glutamate transporter	22.42	6.82E-115	Uhrf1	-11.75	1.24E-111
Fcor	22.41	1.62E-37	Cdc6	-11.67	4.82E-134
AC153955.2	22.33	2.93E-35	Aunip	-11.54	8.99E.73
Fpr1	21.86	3.82E-46	Ccne2	-11.20	5.19E-83
Ifitm1	21.41	5.89E-39	Mgl2	-11.14	1.02E-26
Slc-36a2	20.25	4.14E-44	Mybl2	-11.13	8.67E-161
Naip1	19.04	5.81E-63	Rmi2	-10.89	3.83E-36
Dnah2	18.74	1.89E-15	Exo1	-10.42	1.94E-102
Gpr171	18.51	8.48E-28	Pcaf	-10.42	1.25E-72
Mgst2	16.50	2.85E-57	Chek1	-10.40	1.45E-116

To obtain insight into the underlying mechanisms through which microenvironments regulate macrophage phenotype and function in control and diseased conditions, we analyzed genes uniquely regulated by CO-CM or PH-CM vs. naïve BMDM using Ingenuity Pathway Analysis (IPA). **Figure 1B** shows unsupervised hierarchical clustering and a heatmap generated with these genes. Canonical signaling pathways identified by IPA demonstrated that CO-CM significantly inhibited lipid metabolism, glycolysis, inflammatory pathways, and immune responses (**Figure 1C** left panel) suggesting a metabolically quiescent and anti-inflammatory phenotype. In contrast, when the microenvironment was dictated by disease (i.e. PH-CM), macrophages exhibited distinct upregulated canonical pathways, particularly those related to inflammation (iNOS, Toll-like receptor, NF-kB, TNFR, etc.) and activated immune responses. Pathways involved in metabolism were also notably altered with increased NO and ROS production and decreased oxidative phosphorylation indicating altered mitochondrial function (**Figure 1C** right panel). The complete list of significant canonical pathways regulated by CO-CM and PH-CM is shown in **Supplemental Table 3**.

**Figure 2:**
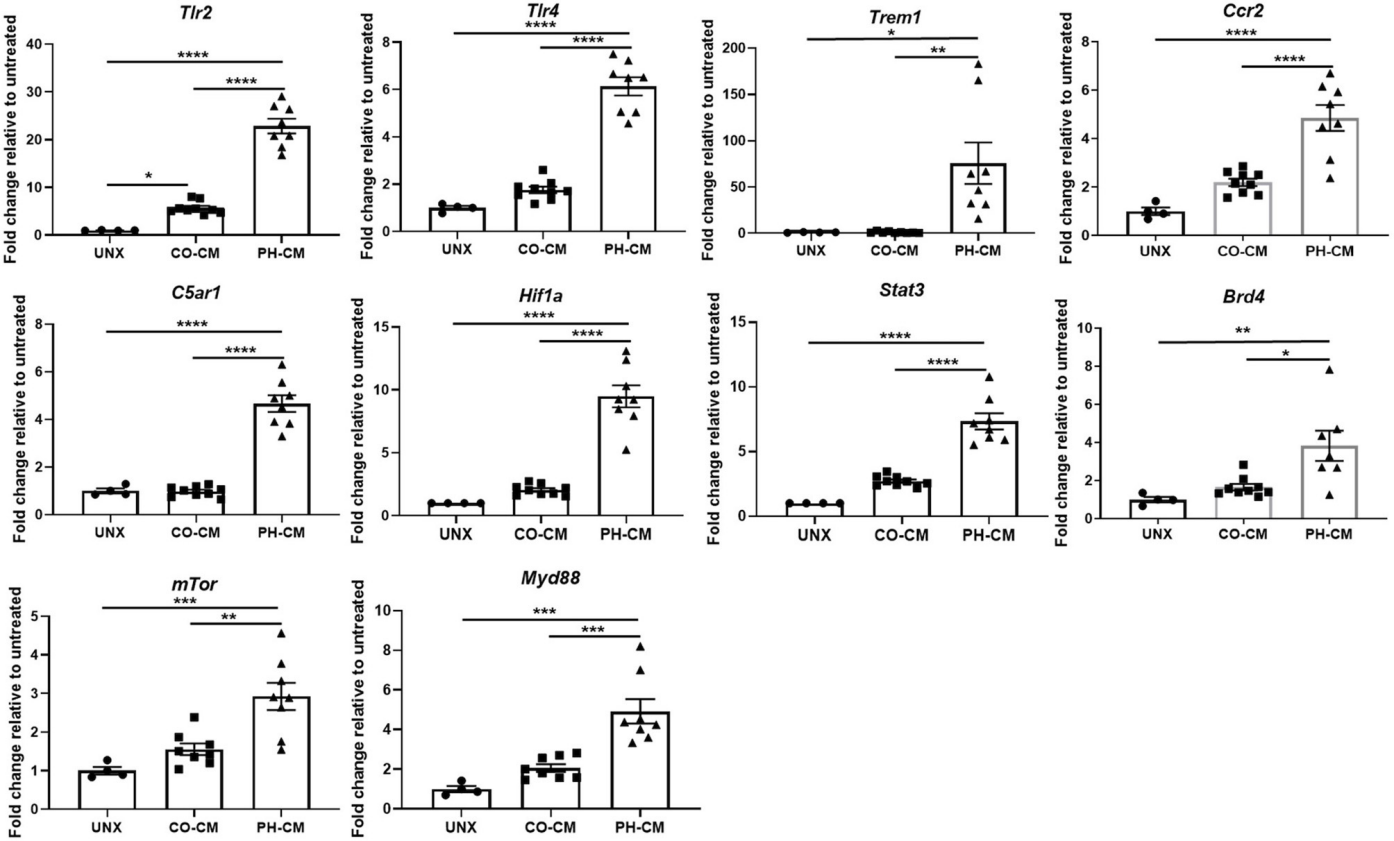
qPCR confirmed gene expression of key upstream regulators in CO-CM and PH-CM stimulated BMDMs. In order to confirm the upstream regulators predicted by IPA in **Figure 1D**. mRNA were collected from untreated (UNX), CO-CM and PH-CM treated BMDMs and gene expression was examined by qPCR analysis. Data is represented by fold change relative to untreated BMDM, and displayed as mean ± sem. *p < 0.05, **p < 0.01, ***p < 0.001, ****p < 0.0001.

We next used IPA to predict Upstream Regulators potentially important for macrophages in response to CO-CM and PH-CM to generate these distinct phenotypes (**Figure 1D**). As defined by IPA, Upstream Regulators are regulatory molecules that may or may not be differentially expressed themselves, but are inferred from clusters of down-stream gene expression. For example, altered activity of transcription factors induced by phosphorylation or translocation may be detected by the expression of down-stream effector genes. The results showed decreased signaling through transmembrane receptors on macrophages (TLR-7 and CCR2) in CO-CM treated BMDMs. Additionally, IPA predicted transcription regulators (STAT3, MYC, RELA, and STAT1) and signaling pathways (mTOR and MYD88) were inhibited by CO-CM in macrophages (**Figure 1D** left panel). On the other hand, the major predicted upstream regulators potentially relevant in the pathophysiological process of PH-CM treated BMDMs are upregulated transmembrane receptors including: 1) TLR2/4 and TREM1 which are triggered by pathogen-associated molecular pattern molecules (PAMPs) or damage-associated molecular patterns (DAMPs); 2) CD40, triggered by signals such as CD40L, TNF and IFN; 3) CCR2, triggered by the chemokine/cytokine CCL2/MCP1; and 4) C5aR1, triggered by complement C5a. Several transcriptional regulators (STAT3, HIF1α, TP53, STAT4, RELA, BRD4, YAP1, NF-kB, CEBP) and signaling pathways (mTOR, MYD88) were also activated as upstream regulators in the presence of PH-CM (**Figure 1D** right panel). The detailed z-scores and p-values of the upstream regulators are listed in **Supplemental Table 4**. We confirmed the gene expression of several of these key upstream regulators in BMDMs by qPCR (**Figure 2**) including transmembrane receptors (Tlr2/4, *Trem1*, *Ccr2*, and *C5ar1*), transcriptional regulators (*Hif1α*, *Stat3*, and *Brd4)*, and cell signaling genes (*mTor* and *Myd88*). We validated these qPCR results using an additional reference gene, 18S, and the results were very similar (**Supplemental Figure 4**). These results underscore the differential regulation of macrophage phenotype by PH versus control fibroblasts.

Previous work in our lab had confirmed a pivotal role for STAT3 in the PH-CM generated activated macrophage phenotype as predicted by IPA analysis in the current study (**Figure 1D**) (19). The current IPA analysis predicts Hif1a to be a critical factor in the induction of the PH-CM generated phenotype. To experimentally evaluate this, we isolated BMDMs from either wild-type or Hif1a knockout mice and treated these BMDMs with PH-CM and analyzed gene expression. We found genetic Hif1a depletion significantly decreased expression of genes that characterize the PH-CM induced macrophage phenotype including *Arg1*, *Il4ra, Socs3*, *Glut1*, and *Vegf* (**Supplemental Figure 5**).

The foregoing analysis utilized naïve mouse BMDMs treated with bovine fibroblast CM because of limited availability of human PA adventitial fibroblasts from PAH patients and control donors. Well-aware of the potential effects attributable to cross-species factors, we sought to confirm and expand these findings by treating bovine BMDMs with bovine fibroblast conditioned media. Finally, as an exploratory tool, we incubated human monocyte-derived macrophages (MDM) with bovine fibroblasts conditioned media. Transcriptional responses to these treatments were compared across all three species. The results (**Supplemental Figure 6**, **Supplemental Table 5**) demonstrated that in general, the gene expression patterns of macrophages/monocytes from mouse, bovine and human in response to bovine fibroblast CM are quite similar. Mouse BMDMs displayed the largest quantitative responses, while bovine BMDMs and human MDM were less responsive. In addition, the trends and quantitative responses of human MDM cells to bovine fibroblast conditioned media are similar.

### Control and Hypertensive Adventitial Fibroblast Conditioned Media Distinctly Regulated Macrophage Cytoplasmic and Mitochondrial Metabolism

The canonical pathway analysis revealed CO-CM and PH-CM have different impacts on macrophage metabolism (**Figure 1C**). We examined key molecules and enzymes involved in metabolism by qPCR and demonstrated that PH-CM induced the expression of genes involved in glycolysis (**Supplemental Figure 7A**, *Glut1, Hk2, G6pd, Pkm2, Ldha, As1*, and *Asl*). Emerging research in PH, similar to other major diseases including cancer, emphasizes the importance of metabolic reprogramming as a determinant of macrophage phenotype in pathophysiological progression (39, 40). Currently the effects of pulmonary vascular adventitial fibroblasts on the metabolic status of macrophages are unknown. In tandem with the observed alterations in metabolic gene transcription, we performed in-depth steady-state metabolomics using UHPLC-MS of CO-CM or PH-CM treated BMDMs, compared to untreated BMDMs.

**Figure 3:**
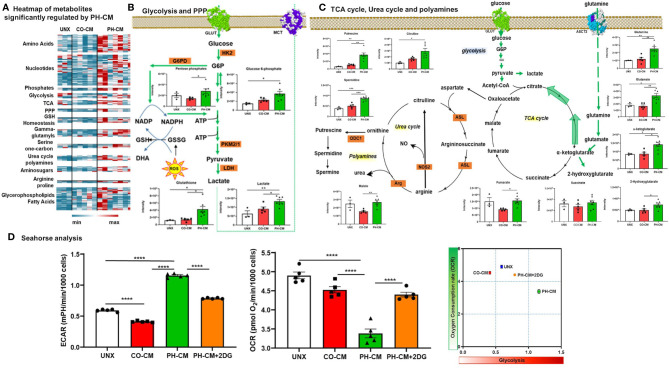
Control and PH-fibroblast conditioned media have distinct effects on regulating macrophages metabolism. **(A)** Heat-map showing metabolites that are significantly regulated by PH-CM compared to CO-CM treated or untreated BMDMs (One-way ANOVA: *p* ≤ 0.05). **(B)** Metabolites in glycolytic or pentose phosphate pathway (PPP) that were significantly increased by PH-CM compared to untreated or CO-CM treated BMDMs. *p < 0.05, **p < 0.01. **(C)** Metabolites in glutaminolysis, TCA cycle, urea cycle or polyamine synthesis that were significantly increased by PH-CM compared to untreated or CO-CM treated BMDMs. *p < 0.05, **p < 0.01, ***p < 0.001. **(D)** CM was collected from CO-Fibs, PH-Fibs or PH-Fibs treated with 2DG. BMDM was exposed to CM for 18hrs prior to their analysis for extracellular acidification rate (ECAR) and oxygen consumption rate (OCR) were measured with Seahorse XF96 analyzer to determine macrophage glycolysis and mitochondrial OXPHOS. ****p < 0.0001.

The heatmap (**Figure 3A**) shows metabolites significantly regulated by PH-CM compared to untreated or CO-CM treated BMDMs. The detailed metabolites are shown in **Supplemental Figure 7B**. In particular, PH-CM increased the accumulation of amino acids, nucleotides, amino-sugars, gamma-glutamyls, glycerophospholipids, and fatty acids in BMDMs compared to untreated or CO-CM treated BMDMs. Glycolytic intermediates including D-glucose-6-phosphate and lactate, the end product of glucose metabolism, and pentose phosphate pathway (PPP) intermediates were increased in PH-CM treated macrophages (**Figure 3B**). Increases in α-ketoglutarate, along with glutamine and glutamate – a substrate for α-ketoglutarate synthesis *via* transamination were also observed. Increases in fumarate and malate but not succinate were noted in PH-CM treated BMDMs. The changes of these metabolites demonstrated an alteration in TCA cycle in response to PH-CM. In addition, we observed increased arginine pathway products, the accumulation of citrulline and polyamines (putrescine, spermidine) in BMDMs incubated with PH-CM (**Figure 3C**). To validate the finding by metabolomic analysis, we measured glycolytic extracellular acidification rate (ECAR) and mitochondrial OXPHOS, represented by oxygen consumption rate (OCR) using Seahorse XF96 analyzer (Agilent). The results showed that compared to untreated BMDMs, CO-CM decreased macrophage glycolysis. In contrast, PH-CM increased glycolysis and decreased oxygen consumption by macrophages. The Glut1 inhibitor, 2DG (10mM), significantly decreased macrophage glycolysis in response to PH-CM (**Figure 3D**). The results from Seahorse analysis confirmed that PH-CM induced metabolic rewiring in macrophages with increase glucose usage and decreased OXPHOS.

### Network Integration Analysis Reveals Coordinate Transcriptional and Metabolic Reprogramming of BMDM by PH-CM.

**Figure 4:**
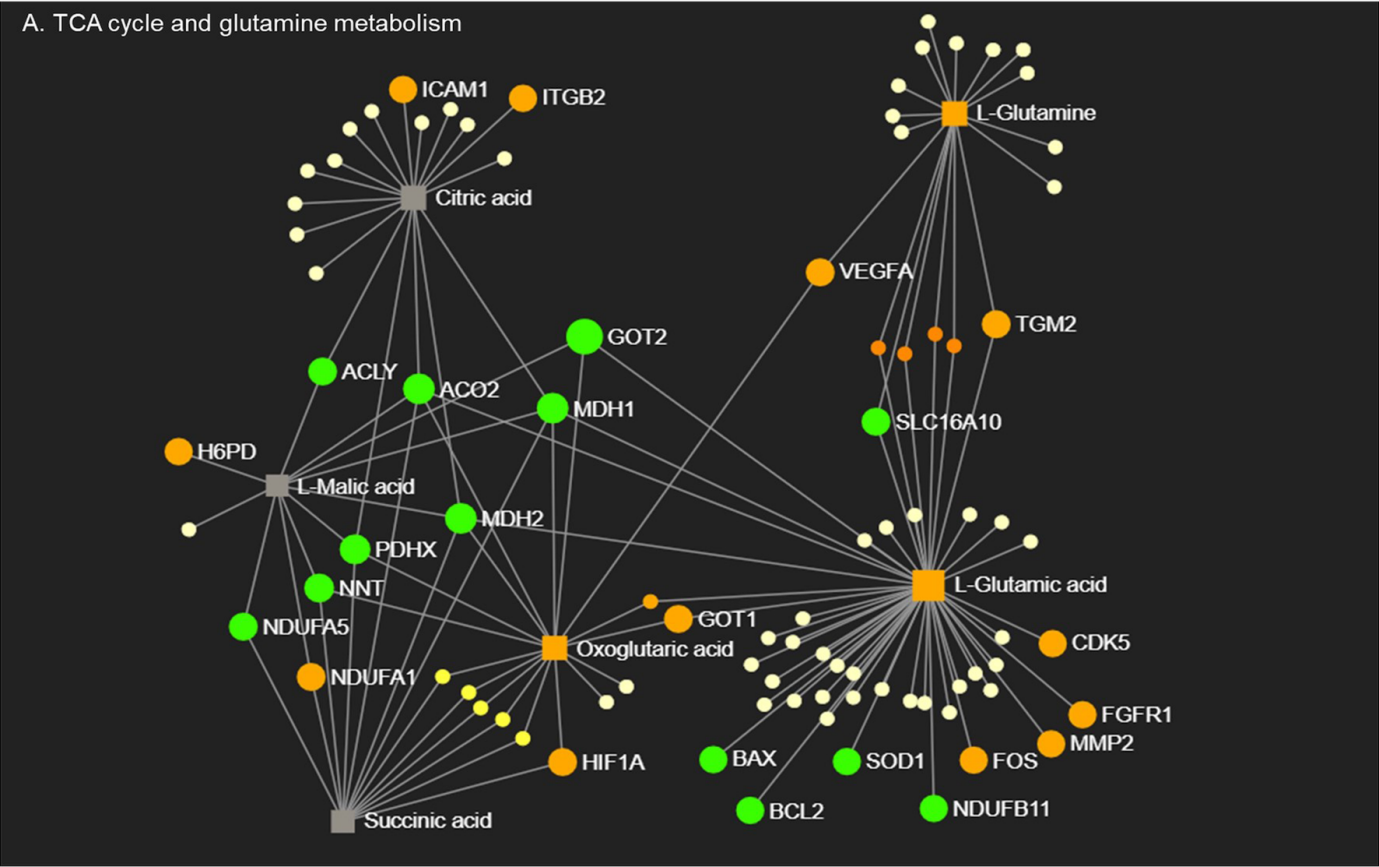
Macrophages respond to PH-CM through an integrated transcriptional and metabolic network. Metabolites in TCA cycle and glutamine metabolism pathways detected in PH-CM treated vs. untreated BMDMs by mass spectrometry were combined with differentially expressed genes (*p* ≤ 0.05) from RNA-seq analysis to generate gene-metabolite network pathways using Metaboanalyst Network Explorer (www.metaboanalyst.ca). Genes and metabolites with established functions in macrophage phenotype are identified and their node symbols are colored based on their levels in PH-CM treated BMDMs compared to untreated BMDMs. Orange circle: increased genes. Green circle: decreased genes. Orange Square: increased metabolites. Grey Square: genes or metabolites not significantly changed in response to PH-CM. Yellow circles: genes or metabolites not known to be significant in macrophage phenotypes. The size of the nodes is arbitrary and does not signify anything.

In order to explore how transcriptional and metabolic mechanisms work together to control macrophage phenotype, we performed network integration analysis combining significantly changed genes and metabolites from RNA-Seq and MS data. CO-CM treated macrophages only generated five sub-networks with limited gene-metabolite interactions (**Supplemental Figure 8A**) whereas PH-CM generated a sophisticated gene-metabolite network (**Supplemental Figure 8B**). We therefore focused on detailed integration analysis of PH-CM treated macrophages. The results could be separated into four key modules (**Supplemental Figure 9**): A) TCA cycle and glutamine metabolism, B) glycerophospholipids and fatty acid metabolism, C) NADH, flavin mononucleotide (FMN), D) arginine, polyamine metabolism, and urea cycle. In particular (**Figure 4**), up-regulated expression of transcriptional regulators (*Hif1a, Fos*) as well as cytosolic and mitochondrial metabolic molecules (*H6pd, Slc16A10, Mdh, Ndufs*) are involved in TCA cycle and glutamine metabolism with associated increased production of L-glutamine, alpha-keto-glutamate in PH-CM activated BMDMs. Correlated gene-metabolite interactions lead to the increased expression of effector genes related to vascular remodeling, extracellular matrix (*Icam1, Itgb2, Vegfa*, and *Tgm2*), growth, cell proliferation (*Cdk5, Fgfr1*) and decreased expression of genes involved in apoptosis (*Bcl2, Bax*). In conclusion, the parallel analysis demonstrated coordinated regulation of macrophage phenotype by PH-CM at both transcriptional and metabolic level.

### The Pro-Inflammatory and Pro-Remodeling Phenotype of BMDMs Treated With PH-Fib Conditioned Medium *In Vitro* Approximate Lung Perivascular Macrophages Isolated From Mice Exposed to Hypoxia *In Vivo*

**Figure 5:**
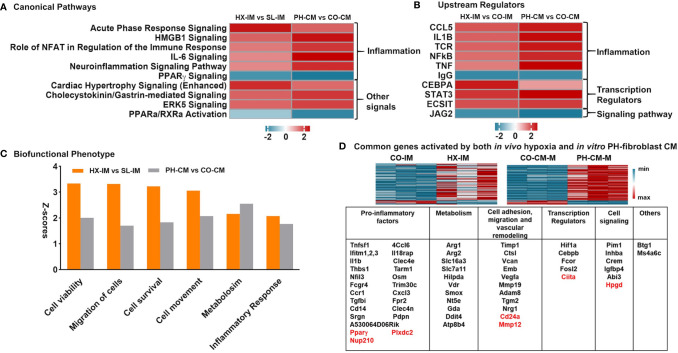
*In vivo* lung interstitial/perivascular macrophages isolated from hypoxic mice and *in vitro* macrophages treated with PH-fibroblast conditioned media share pro-inflammatory and pro-remodeling gene profiles. RNA-seq data of *in vitro* PH-CM treated *vs.* CO-CM treated BMDMs and *in vivo* lung interstitial/perivascular macrophages flow sorted from mouse exposed to 4-day hypoxia (HX-IM) *vs.* sea level (CO-IM) were analyzed. The cutoff criteria is *q* ≤ 0.05 and FC ≥ |2| in order to match the differing data set sizes and to limit our findings to only the most biologically significant pathways. Ingenuity Pathway Analysis identified similarities in: **(A)** Canonical pathways, **(B)** Upstream regulators, and **(C)** Biofunctional phenotypes, part of Disease and Biofunctional profile analysis, with z-score ≥ |2.0| in at least one dataset and *p*-values of overlap ≤ 0.05 in both data sets. **(D)** Common genes significantly regulated by both *in vivo* hypoxia and *in vitro* PH-CM. Genes were clustered in five key categories by their functions: pro-inflammation, metabolism, cell adhesion/migration/vascular remodeling, transcription regulators, and cell signaling. Down-regulated genes are highlighted in red.

Previously, using a hypoxic murine model of PH initiation in combination with flow cytometry assisted cell sorting and RNA-Seq analysis, we demonstrated that lung interstitial/perivascular macrophages (IM) exhibited a “PH program” characterized by mitochondrial dysfunction, pro-inflammatory gene activation and mTOR signaling (13). In order to interrogate how closely our *in vitro* system of PH-CM stimulated mouse BMDMs mimicked the activated interstitial/perivascular macrophage phenotype generated *in vivo*, we compared our previous data from lung interstitial/perivascular macrophages with the current *in vitro* RNA-Seq results. Specifically we determined if similar pathways were generated in these different systems. We used IPA to identify common canonical pathways with z-score ≥ |2| in at least one data set and *p* ≤ 0.05. The results showed similar canonical pathways (**Figure 5A**), predicted upstream regulators (**Figure 5B**), especially those closely related to inflammation, and biofunctional phenotypes (**Figure 5C**) in the macrophages flow sorted from the hypoxic lung and from PH-fibroblast-CM treated BMDMs. RNA-Seq data identified common genes differentially (FC ≥ |2|) regulated by PH-CM *in vitro* and by hypoxia *in vivo* (**Figure 5D**). The majority of these genes can be grouped in five functional groups: inflammatory factors, metabolism, cell adhesion/migration/vascular remodeling, transcription factors, and cell signaling. *IL1b, Thbs1, Slc7a11, Arg2, Vcan* are among the top 30 upregulated genes in PH-CM treated BMDMs (**Table 1**).

### Nicotinamide Adenine Dinucleotide (NADH) and CtBP1 Levels Are Increased in PH-CM Activated BMDMs and Inhibition of CtBP1 Blocks Macrophage Activation by PH-CM *In Vitro*

The integrated gene-metabolite analysis shown in **Supplemental Figure 9** demonstrated a strong NADH related module, and metabolomic as well as Seahorse data clearly indicate that PH-CM stimulated macrophages exhibited marked glycolytic reprogramming. This is reminiscent of our previous work in PH-fibroblasts showing glycolytic reprogramming and increased levels of free NADH, as well as increased activity of CtBP1, a metabolic sensor and transcriptional co-repressor (32). These observations led us to examine whether PH-CM activated macrophage share similar metabolic signaling pathways as PH-Fibs, and thus whether targeting CtBP1 could extend effects on both cell types in the adventitia, restore the adventitial microenvironment toward normal, and be a successful treatment strategy for attenuating macrophage activation *in vitro* and *in vivo*.

**Figure 6:**
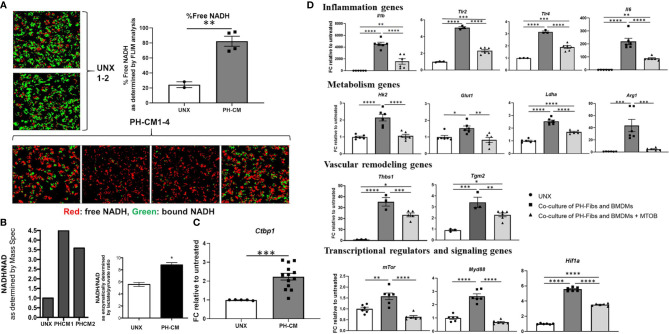
PH-fibroblast conditioned media increased free NADH, as well as CtBP1 levels in BMDMs. MTOB, a small molecule inhibitor of CtBP1, inhibited gene activation in BMDMs in a fibroblast-macrophage co-culture system. **(A)** Fluorescence lifetime imaging (FLIM) of two-photon excited NADH was performed to directly visualize free-NADH (red: arbitrarily colored shorter excitation lifetime, free-NADH. Green: bound-NADH, longer excitation lifetime). A significantly increased detection of free-NADH was seen in PH-CM treated BMDM compared to untreated BMDMs. Data is represented as percentage of red signal and shown as mean ± sem. **p < 0.01 compared to untreated BMDMs. **(B)** NADH/NAD^+^ ratio was measured using Mass Spectrometry analysis and an enzymatic cycling reaction and represented by lactate/pyruvate ratio. *p < 0.05 compared to untreated BMDMs. **(C)** qPCR analysis showed significantly increased *Ctbp1* levels in PH-CM treated compared to untreated BMDMs. Data is represented as fold change relative to untreated BMDMs and displayed as mean ± sem. ***p < 0.001. **(D)** Co-cultured PH-Fibs and BMDMs were treated with MTOB, a small molecule inhibitor of CtBP1. MTOB significantly decreased inflammatory genes *Il1b, TLR2/4, IL6*, metabolic genes *Glut1, Ldha, Hk2, Arg1*, vascular remodeling genes *Tgm2, Thbs1*, and transcriptional regulators and signaling genes *mTOR, MyD88, HIF1a* in co-cultured BMDMs. Data is represented as fold change relative to untreated BMDMs, and displayed as mean ± sem. *P < 0.05, **P < 0.01, ***P < 0.001, ****P < 0.0001.

Fluorescence Lifetime Imaging (FLIM) showed a significant increase of free NADH levels in PH-CM treated BMDMs (**Figure 6A**, red color) compared to untreated BMDMs. NADH/NAD^+^ ratio was also assessed using both Mass Spectrometry and an orthogonal and frequently used method based on an enzymatic cycling reaction and represented by lactate/pyruvate ratio (**Figure 6B**). *Ctbp1* mRNA levels were increased in PH-CM treated BMDMs (**Figure 6C**). These results show that increases in NADH levels and CtBP1 activity are common features of both persistently activated PH-fibroblasts and PH-CM activated macrophages. Our previous work also demonstrated that 4-methylthio-2-oxobutanoic acid (MTOB), a CtBP1 inhibitor, can drive the phenotype of PH-Fibs toward that of CO-Fibs (32). These observations led us to hypothesize that the persistently activated phenotype of fibroblasts and macrophages could be interrupted through treatment with MTOB. We treated PH-fibroblasts and BMDMs in a trans-well system with MTOB. We found decreased expression of genes involved in inflammation (*Il1b, Tlr2, Tlr4*, and *Il6*), metabolism (*Glut1, Ldha, Hk2*, and *Arg1*), vascular remodeling (*Tgm2 and Thbs1*), transcriptional regulators and signaling (*mTor, Myd88, Hif1a*) in BMDMs co-cultured with PH-Fibs in the presence of MTOB (**Figure 6D**).

**Figure 7:**
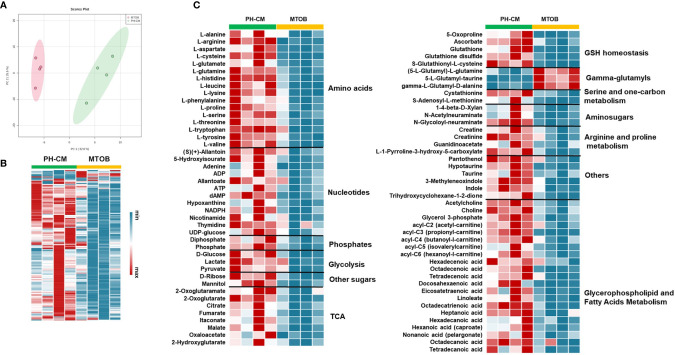
Inhibiting CtBP1 reduced metabolites production in response to PH-CM. **(A)** Principle Component Analysis (PCA) showing clear separation of metabolites produced from BMDMs co-cultured with PH-Fibs with or without MTOB, CtBP1 inhibitor, treatment. **(B)** Heat-map showing in general the concentration of metabolites produced from BMDMs co-cultured with PH-Fibs in the presence of MTOB were decreased compared to in the absence of MTOB. Each column represents an individual sample. **(C)** Heat-map showing metabolites that are significantly changed in BMDMs with MTOB treatment (*p* ≤ 0.05).

**Figure 8:**
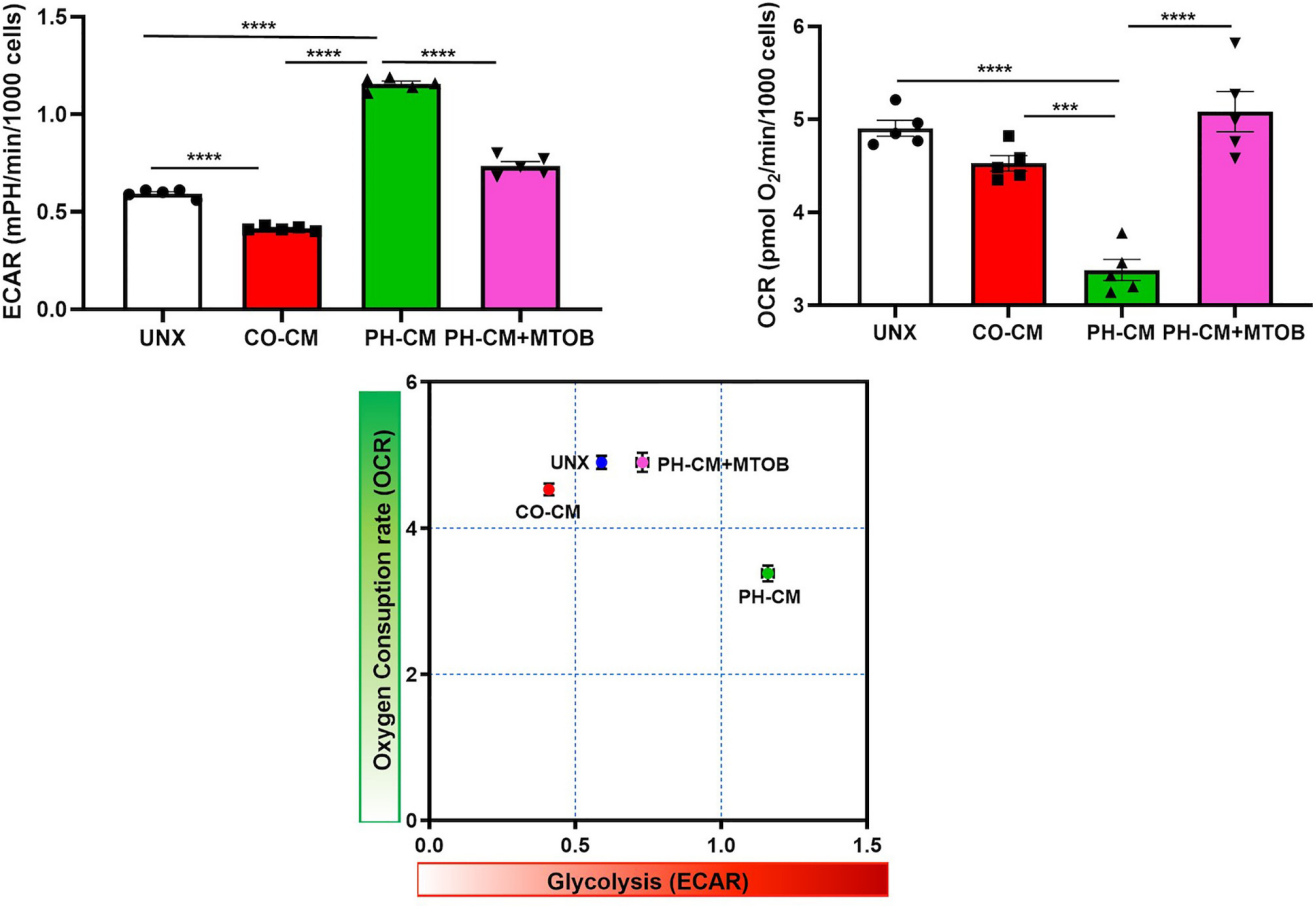
Inhibiting CtBP1 by MTOB reversed the effects of PH-CM on macrophage glycolysis (ECAR) and oxygen consumption (OCAR). CM was collected from CO-Fibs, PH-Fibs or PH-Fibs treated with MTOB. BMDM was exposed to CM for 18hrs prior to their analysis for extracellular acidification rate (ECAR) and oxygen consumption rate (OCR) with Seahorse XF96 analyzer to determine the effects of CtBP1 inhibitor, MTOB, on macrophage glycolysis and mitochondrial OXPHOS. ***p < 0.001, ****p < 0.0001.

We next used UHPLC-MS to examine the effects of MTOB on activated macrophage’s metabolism. PH-Fibs were pre-treated with MTOB, then co-cultured with BMDMs. The overall metabolic profile of macrophages after MTOB treatment is very different from PH-CM activated macrophages (**Figure 7A**, PCA plot, and **7B** heat-map). In particular, MTOB reduced the accumulation of amino acids, nucleotides, amino-sugars, gamma-glutamyls, glycerophospholipids, and fatty acids observed in PH-CM treated BMDMs (**Figure 7C**). Glucose and lactate were decreased by MTOB, glutamine, glutamate, alpha-keto-glutamate were also reduced by MTOB. To extend these findings, we measured extracellular acidification rate (ECAR) and oxygen consumption rate (OCR) with the Seahorse XF96 analyzer to determine macrophage glycolysis and mitochondrial OXPHOS in responses to MTOB treatment. When PH-Fibs were treated with MTOB and that CM transferred to BMDM, we observed significantly decreased glycolysis and increased oxygen consumption compared to PH-CM treated macrophages (**Figure 8**).

### Inhibition of CtBP1 Blocks Hypoxia-Induced Macrophage Accumulation and Activation *In Vivo*

**Figure 9:**
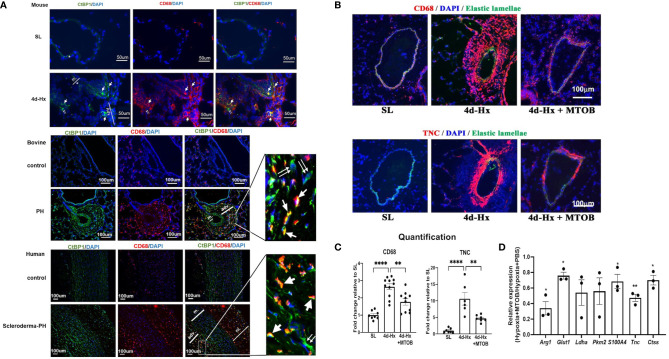
*In vivo* treatment of hypoxia-exposed mice with the CtBP1 inhibitor, MTOB, inhibited lung interstitial/perivascular macrophage activation. **(A)** Immunofluorescent double staining (green: CtBP1, red: CD68, blue: DAPI, scale bar = 50μm for mice and scale bar = 100μm for bovine or human. “m”-media, “adv”-adventitia, “ni”-neointima) demonstrated that CtBP1 protein is overexpressed in the adventitia of 4-day hypoxia-exposed mice, neonatal calves with chronic hypoxia-induced pulmonary hypertension (PH), and human PH patients, but not in their respective controls. Single arrows showed the cells with positive staining for both CtBP1 and CD68. Double arrows showed cells with positive staining for CtBP1 only. **(B)** Immunofluorescent staining of mouse lung sections obtained from sea level (SL) control, 4-day hypoxic (4d-Hx), or 4d-Hx mice treated with MTOB (4d-Hx + MTOB) showed that MTOB decreased inflammatory cell recruitment (CD68) and vascular remodeling (TNC). **(C)** Quantification of CD68 and TNC staining on lung sections from SL, 4d-Hx and 4d-Hx+MTOB mice. **p < 0.01, ****p < 0.0001. **(D)** Lung interstitial/perivascular macrophages were isolated by flow cytometry from mice exposed to hypoxia (HX-IM) and treated with MTOB or PBS. Experiments were repeated three times. qPCR showed MTOB decreased the gene expression of *Arg1, Glut1, Ldha, Pkm2, S100A4, Tnc*, and *Ctss* in interstitial/perivascular macrophages exposed to hypoxia. Data is represented as fold change relative to PBS treated hypoxic mice and is displayed as mean ± sem. *P < 0.05, **P < 0.01.

Given our previous work showing that CtBP1 plays an important role in control of the activated PH-Fib and the current findings supporting a critical for CtBP1 in the PH-CM driven macrophage phenotype, we wanted to explore the possibility that MTOB could potentially act *in vivo* to abrogate reciprocal signalling between the cells and have significant effects on perivascular inflammatory responses. We started by examining CtBP1 expression *in vivo* in hypoxic murine and bovine models, as well as in human PH patients. Co-immunostaining of the macrophage marker (CD68+, red) and CtBP1 (green) of the lung tissues obtained from 4-day hypoxic mice showed higher CtBP1 expression/immunostaining in both CD68^+^ macrophages (single arrows) and CD68^-^ fibroblasts (double arrows) in PA adventitia compared to sea level controls. Similar results were found in both hypoxia-induced PH neonatal calves and human PH patients (**Figure 9A**).

We then tested the therapeutic effect of MTOB *in vivo* in the hypoxic mouse model. It has been reported that hypoxia induced-inflammatory responses peak between day 3 and 4 in mice (2, 13, 32, 41). To determine whether MTOB treatment can prevent hypoxia-induced activation of lung interstitial/perivascular macrophages as predicted by our *in vitro* data, and whether this would abrogate macrophage accumulation and potentially vascular remodeling, we injected mice, exposed to either 4-day hypoxia or kept at sea level, with either MTOB (i.p., 1mg/g) or PBS. We then flow sorted lung interstitial/perivascular macrophages pooled from five mice per group and analyzed their gene expression. The experiment was repeated three times. Immunostaining showed that MTOB significantly reduced hypoxia-induced recruitment of CD68^+^ inflammatory cells in the pulmonary vasculature (**Figures 9B, C**) and confirmed the reduction of PA remodeling as shown by expression of Tenascin C (TNC) (**Figure 9B**). qPCR analysis demonstrated that MTOB treatment significantly decreased macrophage activation marker (*Arg1*) expression, the expression of metabolic (*Glut1)*, inflammatory (*S100a4*), and vascular remodeling genes *(Tnc*, and *Ctss-Cathepsin)* were also decreased in interstitial macrophages flow sorted from hypoxia exposed mice (**Figure 9D**).

## Discussion

Recent studies provide strong support for the hypothesis that in PH, bone-marrow derived monocytes are recruited to the lung perivascular space and differentiate into macrophages (4, 6, 10, 19, 42). Their consistent adventitial/perivascular location has raised the possibility that fibroblasts, the most common cell type in the outer part of the vessel wall, could play a vital role in the recruitment and polarization of recruited monocytes into macrophages with distinct pro-inflammatory, pro-remodeling phenotypes. The intention of this study was twofold: 1) to explore the mechanisms underlying macrophage polarization in response to fibroblast-derived signals in the adventitial microenvironment in both control and PH conditions, and 2) to determine if we could target activation of both fibroblasts and macrophages within the adventitial microenvironment to attenuate the inflammatory responses and vascular remodeling. With regard to the first goal, transcriptomic and metabolomic analysis of naïve mouse BMDMs treated with media conditioned by adventitial fibroblasts isolated from pulmonary hypertensive or age-matched control neonatal calves (PH-CM or CO-CM, respectively) demonstrated the following: 1) PH-CM and CO-CM actively yet differentially regulate macrophage transcriptomic profiles. PH-CM activated inflammation, immune response, and metabolic canonical pathways in BMDMs, while CO-CM inhibited these pathways, 2) the upstream regulators identified in BMDMs treated with PH-CM or CO-CM are distinct and the differences lie in the receptors expressed on BMDMs, as well as activated or inhibited transcriptional regulators and signaling pathways, 3) PH-CM induced metabolic reprogramming in BMDMs characterized by increased aerobic glycolysis, increased PPP, an altered TCA cycle evidenced by increased glutamine utilization, increased polyamine synthesis, and accumulation of amino acids, nucleotides, amino-sugars, gamma-glutamyls, and fatty acids in BMDMs. With regard to our second goal of identifying potential targets common to both fibroblasts and macrophages that if inhibited could reduce inflammation and remodeling, we found: 1) increased glycolysis/free NADH/CtBP1 levels are common features in PH-Fibs and PH-CM treated BMDMs, 2) changing the adventitial microenvironment by targeting this common mechanism with MTOB, a pharmacologic inhibitor of CtBP1, resulted in reduction of metabolite production, and marked inhibition in inflammatory and pro-remodeling gene expression in PH-CM treated BMDMs *in vitro* and significantly attenuated hypoxia induced inflammation and vascular remodeling *in vivo*.

It is well known that macrophage activation is a process that occurs in response to stimuli from the local environment that results in a specific functional phenotype (28, 36, 43–49). The results from our study demonstrated a multi-dimensional macrophage phenotype in the diseased microenvironment associated with metabolic reprogramming in support of their function. **Supplemental Figure 10** illustrates how intracellular metabolic reprogramming of the macrophage can support their function and adaption to the diseased microenvironment. Central to the metabolic reprograming of PH-CM treated macrophages are the increased accumulation of lactate and the alteration of the TCA cycle. Increased levels of lactate and hydroxygluturate, an inhibitor of prolyl-hydroxylases and therefore a HIF stabilizer, both support increased inflammatory gene expression. Increased accumulation of amino acids and nucleotides is the by-product of aerobic glycolysis and a source of energy and building blocks for cell activation and proliferation. Increased flux in the pentose phosphate pathway could also provide substrates for nucleotide synthesis. Additionally, we observed increased levels of glutamine, glutamate, and α-ketoglutarate, suggesting a re-wiring of the fuel preference for the TCA cycle to promote dependence on more amino acid-based sources (e.g. glutamine) rather than glucose derived carbons. PH-CM treated BMDMs also exhibited dramatic accumulation of glycerophopholipids and fatty acids, and increased polyamine synthesis. Collectively this is the first study exploring the effects of adventitial microenvironment on the regulation of macrophage transcription and metabolism. The results suggest that transcriptional and metabolic reprogramming in peri-vascular macrophages could be important in driving many of the hyperproliferative and matrix accumulating phenotypes of cells in the vessel wall. Collectively this is a distinct phenotype that could be considered both pro-inflammatory and pro-remodeling, consistent with the hypothesis that in chronic inflammatory conditions, such as vascular remodeling, there is a non-resolving, co-existence of pro-inflammatory and pro-repair signals. Future studies will need to be directed at determining how this phenotype in early PH evolves over time.

We hypothesized that the vascular remodeling process within the vessel wall of humans and animals with PH entails a bidirectional communication between adventitial fibroblasts and macrophages where products of metabolic programs as well as cytokines produced in activated fibroblasts can induce metabolic reprograming and transcriptional responses in macrophages, and in turn, macrophages provide substrates and factors that regulate fibroblasts. However, at present, we can only successfully study, *in vitro*, one direction of the communication that comprises this hypothesis. We can obtain and maintain in culture phenotypically distinct fibroblasts from control and pulmonary hypertensive vessel walls to examine the effect of these cells on naïve macrophages. Intriguingly, the fibroblasts from the hypoxic vessel wall maintain this distinct phenotype in culture under normoxic conditions. Further, we have shown that exposure of control fibroblasts to hypoxia *ex vivo* is not sufficient to reproduce the PH-Fib phenotype (50). Unfortunately, at present, we cannot derive sufficient numbers of macrophages from the lung vasculature to perform experiments examining the effects of the activated macrophage on the undifferentiated fibroblast. We have preliminary data showing that macrophages treated with PH-Fib conditioned media do exert proinflammatory changes in control fibroblasts. This will be an important question to address in future research.

We were especially intrigued by our observations that the pro-inflammatory and pro-remodeling phenotype of the bone marrow derived macrophages treated with PH-CM *in vitro* resembled the phenotype of lung perivascular macrophages isolated from mice exposed to hypoxia *in vivo*. The phenotype and the underlying signaling mechanisms of interstitial macrophages obtained from hypoxic mice were compared directly to macrophages treated with PH-CM. Both the canonical pathways as well as the upstream regulators were similar between the *in vivo* and *ex vivo* treated macrophages. Upregulation of genes including CCL5, IL-1beta, and IL-6 and upstream regulators including STAT3, HIF-1, and NFkB and a striking decrease in PPAR gamma were observed both *in vivo* and then *ex vivo* treated macrophages. These observations support the idea that the robust and important macrophage phenotypes observed *in vivo* can potentially be recapitulated *in vitro*, at least in its initial stages, and used to gain insight into the critical signaling mechanisms in this very distinct macrophage that contributes to PH.

Pulmonary hypertension is a disease characterized by chronic and persistent inflammation. Fibroblasts and macrophages are the primary cell types in the adventitial area, the hub for inflammatory signaling. The cross-talk between fibroblasts and macrophages, as well as the microenvironment harboring these cells facilitates the chronic inflammation observed in PH. Interestingly, we observed common phenotypes and common underlying regulatory mechanisms in PH-fibroblasts and PH-CM treated macrophages. Increases in aerobic glycolysis, free NADH, and activity of the transcriptional co-repressor, CtBP1, were observed in both cell types. Increases in CtBP1 are particularly interesting as this transcriptional co-repressor has also been reported to contribute to the metabolic and phenotypic abnormalities in cancer and microglia cells (38, 51, 52). Previous studies demonstrated inhibition of CtBP1 with MTOB abrogated the activated phenotype of PH-Fibs and reversed the metabolic profile of PH-Fibs toward CO-Fibs. In this study, we treated PH-Fibs and macrophages that were in co-culture with MTOB and reduced both macrophage inflammatory gene expression and metabolite production. This raised the possibility that we could potentially abrogate the cross-talk between these two cells *in vivo*. We treated hypoxic mice with MTOB and found decreased inflammatory cell accumulation, as well as decreased expression of an early marker of vascular remodeling, Tenascin C, in the lung perivascular area consistent with previous observations (32). In flow sorted macrophages from the MTOB treated hypoxic mice, we found significant decreases in markers associated with metabolism, inflammation and extracellular matrix supporting the idea that targeting metabolism in cell types driving chronic inflammation could abrogate inflammatory responses. This is consistent with observations in various cancers wherein CtBP has evolved as a potential therapeutic target in cells expressing a glycolytic phenotype (51, 53). One recent study found the emergence of cells (stem-cell like breast cancer cells) with resistance to metformin treatment but with heighted sensitivity to inhibitors of CtBP. Thus, a better understanding of metabolic transcriptomic cross-talk in PH may lead to identification of novel metabolic targets.

In conclusion, the results from our study provide the basis for a more systematic mechanistic understanding of the cross-talk between adventitial fibroblasts and macrophages in health and disease by revealing a comprehensive view of the macrophage transcriptomic and metabolomic landscape. Furthermore, phenotypic and regulatory similarities between PH-Fibs and BMDMs exposed to PH-CM offer an opportunity for novel therapies targeting a coordinated signaling unit. Such therapeutic approaches offer the exciting possibility of attenuating or reversing the pathophysiological remodeling which drives PH progression, but is not altered by current vasodilator therapies.

## Data Availability Statement

The RNA-seq data presented in this article have been submitted to the National Center for Biotechnology Information Gene Expression Omnibus repository (GEO) (http://www.ncbi.nlm.nih.gov/geo/). The GEO accession number is GSE165500.

## Ethics Statement

All animal procedures were performed in accordance with the guidelines for animal experimentation established and approved by the Institutional Animal Care and Use Committees of the University of Colorado Anschutz Medical Campus and Colorado State University (mice and neonatal calves, respectively). All human tissue samples were de-identified and were used secondarily after their primary collection purpose. Pittsburgh University Tissue Bank has obtained permission to study the tissues obtained.

## Author Contributions

ML, SR, KEK, and KS conceived the study. ML, SR, HZ, JP, AM, ADA, MF, MO, TN, MF, SK, and AL carried out the experiments and analyzed the data. ML, SR, DB, SK, and KS and wrote the paper. All authors contributed to the article and approved the submitted version.

## Funding

NIH/NHLBI R01HL125827, NIH/NHLBI P01HL152961, NIH/NHLBI T32HL007171, DOD W81XWH1910259, DOD W81XWH2010249, ADA: Boettcher Webb-Waring Award.

## Conflict of Interest

The authors declare that the research was conducted in the absence of any commercial or financial relationships that could be construed as a potential conflict of interest.
