# Supplementary material for: Microenvironmental Regulation of Macrophage Transcriptomic and Metabolomic Profiles in Pulmonary Hypertension
Source: Front Immunol. 2021 Mar 31;12:640718. doi: 10.3389/fimmu.2021.640718 (PMC8044406; doi:10.3389/fimmu.2021.640718)
Supplement: Supplementary file 1 [file DataSheet_1.docx]

Supplementary Material

**COMPLEMENTARY DETAILS OF MATERIALS AND METHODS**

**Animals**

All animal procedures were performed in accordance with the guidelines for animal experimentation established and approved by the Institutional Animal Care and Use Committees of the University of Colorado Anschutz Medical Campus and Colorado State University (mice and neonatal calves, respectively). The neonatal calf model of severe chronic hypoxia-induced PH has been described previously (1-3). Briefly, neonatal male Holstein calves (1-3 days old) were exposed to hypobaric hypoxia (P_B_ = 430mmHg, simulated elevation 15,000 ft. / 4,570 m) for 2 weeks, while age-matched controls were kept at ambient Fort Collins, CO altitude (P_B_ = 640 mmHg, 5,000 ft. /1,520 m). Standard husbandry and veterinary care were provided following institutional guidelines at the Hypobaric/Hyperbaric Facility, Department of Physiology, Colorado State University (Fort Collins, CO). Following this interval, animals were deeply anesthetized with sodium pentobarbital (160mg/kg) and exsanguinated for tissue harvest.

All mice were C57bl6/J background. Wildtype and *Hif1α*^fl/fl^;LysMcre were purchased from Jackson Laboratories (Bar Harbor, Maine, USA). Mice were kept at simulated sea level altitude to offset Denver altitude for one week after arrival with controlled temperature (22–24 °C) under a 12-hour-light-dark cycle. Food and water were accessible ad libitum. After one week, mice were randomly separated into three groups (n=5/each group): 1) sea level normoxia, 2) hypoxia + PBS, and 3) hypoxia + 4-methylthio-2-oxobutanoic acid (MTOB, Sigma-Aldrich, Saint Louis, MO; I.P injections, 1mg/g body weight. Mice were pre-treated for one-week, 3 times per week, at sea level before hypoxic exposure, and injected once while under hypoxic exposure). Hypoxic groups were placed in hypobaric hypoxic chambers (P_B_ =380mmHg, simulated altitude of 18,000ft/5,490 m) for 4 days, corresponding to the peak of macrophage recruitment and inflammation observed previously (4-6). Hypoxic exposure was supervised using an oxygen monitor. Control mice (normoxia) remained at sea level altitude under the same light-dark cycle.

**Cell Culture**

Adventitial fibroblasts from distal pulmonary arteries (dPAs) of 2-wk old male calves (control or with hypoxia induced PH) were isolated by explant culture as described previously (2, 7, 8) and were cultured with 10% Bovine Calf Serum in DMEM media. Human pulmonary artery fibroblasts were derived from patients with idiopathic PH or from control donors undergoing lobectomy or pneumonectomy and were cultured with 10% Fetal Bovine Serum. All cells were cultured under ambient (Denver) normoxic conditions. Experiments were performed on cells between passages 5-10. Conditioned media (CM) was collected after 1 day of growth when the cells were greater than 85% confluent. After collection, CM was centrifuged to remove any cell debris, and stored at -80°C prior to experimental use.

Mouse bone marrow-derived macrophages (BMDMs) were prepared by incubating extracted bone marrow from male wild-type or *Hif1α*^fl/fl^;LysMcre C57bl6/J mice in “Macrophage Growth Medium”, DMEM media containing 2mM glutamine, 25mM HEPES, 1mM pyruvate supplemented with 10% FBS (Endotoxin levels below 0.3 IU) and 50ng/ml CSF1, for 5-10 days. For CM treatment experiments, 0.5 million BMDMs were plated per well of a 24well dish. BMDMs were treated with fibroblast CM supplemented with 10%FBS for 18 hours for RNA isolation (9). The effect of MTOB treatment on macrophage was assessed in a co-culture system. PH-fibs were either left untreated or pre-treated with CtBP1 inhibitor, MTOB. Prior to exposing BMDM to pretreated PH-Fibs CM, the media was supplement with 1/3 final volume of fresh media, with or without drug. The BMDM were exposed to PH-Fibs through a Transwell system for 18hr prior to harvest.

Bovine BMDM were propagated from cells isolated from the ribs of control neonatal calves. Freshly isolated bovine bone marrow cells are incubated in growth media [10%FBS, DMEM (with pyruvate and 25mM Hepes) + Non-Essential Amino Acids + 10mM Gln + 50ng/mL human CSF1] for 9-14 days until a confluent culture of macrophage cells is achieved. Alternatively, freshly isolated bone marrow cells are cryopreserved in FBS: DMSO (9:1 v:v). Cells are revived from frozen stock by dilution in DMEM media followed by centrifugation at 300x*g* for 5 min and cultured by the same method as freshly isolated bone marrow cells.

Human monocyte-derived macrophages (MDM) were derived from monocytes isolated from human peripheral blood mononuclear cells (PBMC) using the Miltenyi Human Pan Monocyte Isolation Kit, 130-096-537. PBMC were provided by the Healthy Human Blood Cell Research Consortium at National Jewish Health, Denver, CO after purification from whole peripheral blood using a plasma Percoll method as previously described (10) and washed 2 times using Krebs-Ringer phosphate-buffered saline with dextrose (154 mM NaCl, 5.6 mM KCl, 1.1 mM MgSO_4_, 2.2 mM CaCl_2_, 0.85 mM NaH_2_PO_4_, 2.15 mM Na_2_HPO_4_ and 0.2% dextrose). A half of a million monocytes were plated per well of a 24 well plate and cultured for a week in DMEM (Corning 15-018) supplemented with 4mM glutamine, pyruvate, penicillin-streptomycin, 10% FBS (endotoxin levels <0.3EU), and 100ng/mL human CSF (Bio-Legend, 574806). After one week of culture, the MDM were stimulated with either conditioned media derived from CO-Fibs or PH-Fibs and supplemented with 10%FBS for 18hr prior to harvest for analysis.

**Mouse Lung Interstitial/Perivascular Macrophage Preparation and Flow Cytometry**

The hypoxic mouse model is well established in our laboratory (11, 12). Mice were exposed to hypoxia or sea level environments as described above. For isolation of lung interstitial/perivascular macrophages (IM), five mice were pooled per sample due to limited cell yields. The experiment was repeated three times for each group. Flow cytometry analysis and cell sorting were conducted at the University of Colorado Cancer Center Flow Cytometry Core Facility as described previously (12). Briefly, interstitial/perivascular macrophages were isolated by flow cytometry from total lung digest using the following gating strategy: Debris, doublets and dead cells were excluded, cells were then sorted against pre-injected intravenous CD45 antibody to remove intravascular cells, followed by sorting on CD64 and side scatter to identify macrophages, and then interstitial/perivascular macrophages were separated from alveolar macrophages by high-CD11b/low-CD11c vs. high-CD11c/lowCD11b, respectively. In the 4-day hypoxic mouse, the interstitial macrophage population was confirmed to be primarily perivascular as shown by macrophage immunostaining in whole lung sections (4, 12).

**RNA Sequencing (RNA-Seq)**

Mouse BMDMs were treated with media conditioned by either CO-Fibs (CO-CM) or PH-Fibs (PH-CM) or left untreated (UNX). Each treatment CM was generated from CMs pooled from 5 different fibroblast populations. The experiment was repeated three times. Total RNA was purified using NucleoSpin RNA Isolation kit (Machery-Nagel, [www.mn-net.com](http://www.mn-net.com)) according to the manufacturer’s instructions. RNA quantity and quality were evaluated using NanoDrop and Bioanalyzer. The RNA Integrity Number (RIN) values were near 10 in all the samples. RNA-Seq library preparation and sequencing were conducted at the Genomics and Microarray Core at the University of Colorado Anschutz Medical Campus. Libraries were constructed using a NuGen universal plus mRNA-Seq kit. Sequencing was performed on Illumina NovaSEQ6000 system, using the paired-read 2x150 cycle option.

**Bioinformatics Analysis**

RNA-Seq reads were generated using Illumina NovaSEQ6000 analysis pipeline. In this study we also included our previously published interstitial macrophage raw data (12), archived at <https://www.ebi.ac.uk/ena/data/view/PRJNA345360>. The read quality of all the samples were checked using FastQC v0.11.5. Adapter trimming, quality control, and base correction were performed by AfterQC (13). The sequencing reads in the FASTQ files of all the samples were aligned to the Mus musculus reference genome (Mus_musculus.GRCm38v91) (14) with Spliced Transcripts Alignment to a Reference (STAR) version 2.6.0c (15). STAR is an ultrafast universal RNA-Seq aligner that specifically addresses RNA-Seq data mapping challenges using strategies that makes it faster and more accurate than other current alternatives. The RNA-Seq FASTQ reads were aligned using the following STAR parameters: --genomeDir INDEX; --sjdbGTFfile GTF; --runThread 16 ; --outSAMstrandField intronMotif ; --outFilterIntronMotifs RemoveNoncanonical ; --readFilesIn fastq1 fastq2 ; --readFilesCommand zcat ; -- outSAMtype BAM Unsorted ;--outReadsUnmapped Fastx ;--outSAMmode Full. We next applied featureCounts v1.6.2 (parameters used: -T 16; -t exon; -g gene_id; -a GTF; bam) (16) to assign reads to genes using mouse transcript annotations as a guide.

EdgeR (version 3.14) in R (version 3.4.3) was used for data normalization and differential expression analysis of RNA-Seq expression profiles with biological replication (17). Briefly, it implements a range of statistical methodologies based on the negative binomial distributions for both biological and technical variability. Additionally, it uses an empirical Bayes method to moderate the degree of over-dispersion across transcripts, thereby improving the reliability of inference. A modification of the Fisher exact test was used as the default method to determine differential gene expression for pairwise comparisons. The Benjamin-Hochberg procedure (18) was used to control the false discovery rate (**FDR**), and a cut-off criterion of FDR ≤ 0.05 was applied to identify differentially expressed genes. Individual gene expression was calculated as ‘reads per kilobase per million mapped reads’ (RPKM). Finally, differentially expressed genes were selected based on fold-change (≥ |1.2|), and FDR value (*q* ≤ 0.05). Heat-map and hierarchical clustering were performed using a bioconductor package ComplexHeatmap v1.10.2 (19). Gene expression levels were centered on the mean of log transformed RPKM. Clustering was done using complete linkage and Euclidean distance method. Gene ontology and signaling network analysis was performed with Ingenuity Pathway Analysis (IPA, [www.ingenuity.com](http://www.ingenuity.com)).

As shown in Supplemental Fig 1., of the 53,465 genes sequenced, 12,191 met quality control standards and with count greater than 1. 3,348 genes were further removed because neither CO-CM nor PH-CM significantly regulated the expression of these genes compared to untreated BMDMs (Cutoff criteria for differential gene expression: fold change ≥ |1.2|, and FDR ≤ 0.05). The rest of differentially expressed genes were shown in VENN diagrams. Both CO-CM and PH-CM treatment significantly altered expression of a shared subset of 5,924 genes. Among these genes, there are 5,756 genes were regulated in the same direction (either up- or down-regulated) by both CO-CM and PH-CM. The remaining 168 genes were regulated to the opposite directions by CO-CM or PH-CM. There are 1,061 genes significantly regulated by CO-CM only, 1,858 genes were significantly regulated by PH-CM only. The uniquely regulated genes (indicated by pentagons) by CO-CM (1,061+168=1,229) and PH-CM (1,858+168=2,026) were used for pathway analysis.

**Quantitative RT-PCR**

Total RNA of cultured cells was isolated using the NucleoSpin RNA Isolation kit (Machery-Nagel, [www.mn-net.com](http://www.mn-net.com)) according to the manufacturer’s instructions. Total RNA from wild-type mouse lung interstitial/perivascular macrophages was isolated post flow cytometric sorting using a hybrid of Trizol (Ambion) extraction and Qiagen RNeasy MinElute clean up kit (Qiagen) as follows. Cells were lysed in Trizol, Chloroform was added in a 1:5 ratio, pelleted at 12,000xg for 15 minutes, aqueous layer removed and combined 1:1 with 100% ethanol, and placed into Qiagen MinElute column. RNA was further cleaned up per RNeasy MinElute protocol (Qiagen). RNA quality and quantity were analyzed using a NanoDrop and Bioanalyzer. First-strand cDNA synthesis was performed using an iScript cDNA Synthesis Kit (Bio-Rad, Hercules, CA). Quantitative Real-Time PCR was performed using either TaqMan probes (Applied Biosystems, Grand Island, NY) or SybrGreen primers (Suppl. Table 1) according to the manufacturer’s instructions. Gene expression was calculated after normalization to *Hprt* using the ΔCt method and the fold change was calculated relative to the controls in each group.

**Ultra-High Pressure Liquid Chromatography-Mass Spectrometry-Based Metabolomics Analyses**

Metabolomics analyses were performed as previously reported (4, 20) with minor modifications. There are two metabolomics analyses in this study. The first analysis was done with BMDMs treated with CO-CM, PH-CM, or left untreated for 18hrs. The second analysis was done with BMDMs in co-culture with PH-Fibs. After treatment, cells were pelleted and lysed at a concentration of 3 x 10^6^ cells/ml in ice-cold buffer (methanol/acetonitrile/water, 5:3:2, v/v/v) for analysis. The analytical platform employs a 5 minute C18 gradient on a Vanquish UHPLC system coupled to a Q Exactive mass spectrometer (Thermo Scientific, Bremen, Germany) in positive and negative ion modes (separate runs) (21). NAD and NADH were separated on a Kinetex C18 column (2.1 x 150 mm, 1.7 um) using a 9 min gradient. Raw files were converted to mzXML using MassMatrix and peak areas extracted in Maven using QC measured. Metabolites concentrations were normalized by protein content of each sample.

**Integration of Transcriptional and Metabolic Data**

RNA-Seq and Mass Spectrometry data of BMDMs treated with CO-CM and PH-CM were analyzed together using MetaboAnalyst (<https://www.metaboanalyst.ca/>) (22). All differentially expressed genes and metabolites after treatment with conditioned media were uploaded to generate overall interaction networks. For specific metabolite modules, all metabolites detected in the specific pathway were uploaded regardless their differential expression and analyzed with all differentially expressed genes to create a more complete network.

**Measurement of glycolysis and Oxygen Consumption Rate by Seahorse Analysis**

PH-Fibs were treated with a glycolysis inhibitor, 2-DG (10mM), for 24hrs, or CtBP1 inhibitor, MTOB (2.5mM), for 48hrs. CO-CM, PH-CM, as well as 2DG or MTOB treated PH-CM (PH-CM+2DG, PH-CM+MTOB, respectively) were collected as described above, stored in -80°C until use. The Seahorse Bioscience XF96 Extracellular Flux analyzer (Agilent, North Billerica, MA, USA) was used to evaluate the mitochondrial respiration of BMDMs by measurement of oxygen consumption rate (OCR) and extracellular acidification rate (ECAR). These studies were supported in part by the Molecular and Cellular Analytical Core in the Colorado Nutrition Obesity Research Center (P30 DK48520). Briefly, mouse BMDMs were seeded in a 96-well Seahorse plate at a density of 8x10^4 cells/well and were incubated with 80ul of different conditioned medium or left untreated. After overnight incubation, media were changed to unbuffered Seahorse XFDMEM medium, pH7.4 supplemented with 10mM glucose and 1mM sodium pyruvate for 1hr. Then, OCR and ECAR were measured under basal conditions according to the manufacturer's instructions. The results were normalized for cells numbers and analyzed using the Wave Software (Agilent) (23, 24).

**Fluorescence Lifetime Imaging (FLIM)**

Mouse BMDMs were cultured as described above and treated with PH-CM for 24hrs prior to measurement of intracellular NADH level by fluorescence lifetime imaging (FLIM). FLIM of two-photon excited NADH was performed at the UCD Advanced Light Microscopy Core to directly quantitate NADH level in cells as previously described (4, 25-28). Even though the fluorescence excitation and emission properties of the nicotinamide ring of NADPH are identical to those of NADH, it was demonstrated that FLIM can separate free-NADH and NADPH fluorescence lifetimes in live cells and tissues. Another important consideration is that the dissociation constant (Kd) of CtBP is much higher for NADH (1nM) than NADPH (10^4nM), which indicate that the relative contribution of free NADPH to CtBP activation would have been minimal (25, 26, 28).The FLIM experiments were performed in the Advanced Light Microscopy Core part of the NeuroTechnology Center at University of Colorado Anschutz Medical Campus supported in part by Rocky Mountain Neurological Disorders Core Grant Number P30 NS048154 and by Diabetes Research Center Grant Number P30 DK116073. Contents are the authors' sole responsibility and do not necessarily represent official NIH views.

**Assessment of NADH/NAD+ Ratio**

NADH/NAD+ ratio was determined using modified methods of DH (29) based on the enzymatic cycling reaction (Pyruvate + NADH + H+ ↔ Lactate + NAD+ ) and was performed as previously described (4, 28). Briefly, cells were cultured in 60 mm Petri dishes and exposed to conditioned media or left untreated. After 18hrs, cells were washed with cold PBS and homogenized with 100 µl of cold acid-extraction buffer (1 M HClO3) and then neutralized with 50 µl of cold 2 M KHCO3. The pH of HCLO3 and KHCO3 mixture was 7.0. The absorbance of NADH at 340nM was measured before and after an enzymatic cycling reaction. The difference of NADH absorbance was used to determine the concentration of lactate or pyruvate. Standard curves for lactate and pyruvate concentration were generated for every set of experiments. The ratio of [lactate]: [pyruvate] was used to calculate the NADH:NAD+ ratio according to the following equation: NADH: NAD+ = K(lactate)/(pyruvate)(H+ ), where K is the equilibrium constant for lactate dehydrogenase.

**Immunostaining Staining**

Freshly obtained animal lung tissue samples were embedded in O.C.T. (Sakura FineTek, Torrancve, CA) and frozen at -80°C. For indirect immunostaining (single- or double-labeling), 5 μm tissue cryosections were fixed in cold methanol:acetone (1:1).Nonspecifc binding was blocked with DAKO Antibody Diluent (DAKO Inc.,Carpinteria,CA),and sections were incubated overnight at 4°C with the following primary antibodies (Abs): mouse monoclonal Abs (mAbs) against mouse CtBP1 were purchased from Epigentek (Farmingdale, NY) and used at 1:100 dilution. Mouse mAbs against bovine and human CtBP1 were purchased from BD Biosciences (San Jose, CA), and used at 1:100 dilution. Rat mAbs against mouse macrophage marker CD68 were purchased from Bio-Rad (Hercules, CA) and used at 1:400 dilution. Mouse mAbs against bovine and human macrophage marker CD68 were purchased from DAKO (Raleigh, NC) and used at 1:200 dilution. Rabbit polyclonal Abs against tenascin C (TNC) were purchased from EMD Millipore (Billerica, MA. Brand Family: Chemicon), and used at 1:100 dilution. Single-labeling was performed via a Biotin-Streptavidin detection system using Alexa-488 or -594 fluorochromes according to manufacturer’s instructions (Molecular Probes/Invitrogen, Frederick, MD). For double-labeling, appropriate species-specific biotinylated Abs were used as secondary Abs,followed by Streptavidin-conjugated fluorochrome. Immunolabeled sections were mounted with VectaShield embedding medium with DAPI (Vector Labs, Burlingame, CA), and examined under a Zeiss fluorescent microscope. Images were acquired using AxioVision digital imaging system.

**Quantification of Immunofluorescent Staining**

5 μm tissue sections with immunofluorescent staining were scanned on a Leica-Aperio Versa 8 system at 20x magnification. The acquired images were then analyzed using the Aperio ImageScope software v12.4.2 with the Area quantification FL v1 algorithm. The red fluorescence threshold was selected to eliminate the out-of-fluorescence background. The entire lung lobe section area was selected for quantification, and the red fluorescence was normalized to the total lung area. Two lung sections per each group were analyzed. The resulting ratio was presented as “Expression” in Artificial Units (AU). The data was presented as fold change relative to sea level mouse lung section.

**Human Tissues**

Human pulmonary artery specimens from rejected normal donors (n=3) and patients with pulmonary (arterial) hypertension (PH/PAH) (n=5) (see Suppl. Table 2 for detailed description of clinical characteristics, demographics and pathological diagnoses) were obtained from Pittsburgh University Tissue Bank. Samples were de-identified and were used secondarily after their primry collection purpose. Pittsburgh University Tissue Bank has obtained permission to study the tissues obtained.

**Statistical Analysis**

Prism software version 7 (GraphPad, Software, Inc, [www.graphpad.com](http://www.graphpad.com)) was used for t-test or one-way ANOVA followed by Bonferroni post-test analysis for multiple comparisons. Values were expressed as mean ± SEM. For basic comparisons of two Gaussian distributed sample sets, we used Student’s unpaired, two-tail t-tests. When comparing multiple groups, the respective ANOVA (one way when comparing one characteristic, or two way if two dependent variables were involved) was performed. P-values were subject to multiple testing adjustment using Bonferroni correction. Differences with *p* values less than 0.05 were considered statistically significant.

**SUPPLEMENTAL REFERENCES**

1. Das M, Burns N, Wilson SJ, Zawada WM, Stenmark KR. (2008). Hypoxia exposure induces the emergence of fibroblasts lacking replication repressor signals of PKCzeta in the pulmonary artery adventitia. Cardiovasc Res. 78:440. doi:10.1093/cvr/cvn014

2. Li M, Riddle SR, Frid MG, El Kasmi KC, McKinsey TA, Sokol RJ, et al. (2011). Emergence of fibroblasts with a proinflammatory epigenetically altered phenotype in severe hypoxic pulmonary hypertension. J Immunol. 187:2711. doi:10.4049/jimmunol.1100479

3. Stenmark KR, Fasules J, Hyde DM, Voelkel NF, Henson J, Tucker A, et al. (1987). Severe pulmonary hypertension and arterial adventitial changes in newborn calves at 4,300 m. J Appl Physiol (1985). 62:821. doi:10.1152/jappl.1987.62.2.821

4. Li M, Riddle S, Zhang H, D'Alessandro A, Flockton A, Serkova NJ, et al. (2016). Metabolic Reprogramming Regulates the Proliferative and Inflammatory Phenotype of Adventitial Fibroblasts in Pulmonary Hypertension Through the Transcriptional Corepressor C-Terminal Binding Protein-1. Circulation. 134:1105. doi:10.1161/CIRCULATIONAHA.116.023171

5. Paddenberg R, Stieger P, von Lilien AL, Faulhammer P, Goldenberg A, Tillmanns HH, et al. (2007). Rapamycin attenuates hypoxia-induced pulmonary vascular remodeling and right ventricular hypertrophy in mice. Respir Res. 8:15. doi:10.1186/1465-9921-8-15

6. Vergadi E, Chang MS, Lee C, Liang OD, Liu X, Fernandez-Gonzalez A, et al. (2011). Early macrophage recruitment and alternative activation are critical for the later development of hypoxia-induced pulmonary hypertension. Circulation. 123:1986. doi:10.1161/CIRCULATIONAHA.110.978627

7. Frid MG, Li M, Gnanasekharan M, Burke DL, Fragoso M, Strassheim D, et al. (2009). Sustained hypoxia leads to the emergence of cells with enhanced growth, migratory, and promitogenic potentials within the distal pulmonary artery wall. Am J Physiol Lung Cell Mol Physiol. 297:L1059. doi:10.1152/ajplung.90611.2008

8. Wang D, Zhang H, Li M, Frid MG, Flockton AR, McKeon BA, et al. (2014). MicroRNA-124 controls the proliferative, migratory, and inflammatory phenotype of pulmonary vascular fibroblasts. Circ Res. 114:67. doi:10.1161/CIRCRESAHA.114.301633

9. El Kasmi KC, Pugliese SC, Riddle SR, Poth JM, Anderson AL, Frid MG, et al. (2014). Adventitial fibroblasts induce a distinct proinflammatory/profibrotic macrophage phenotype in pulmonary hypertension. J Immunol. 193:597. doi:10.4049/jimmunol.1303048

10. Haslett C, Guthrie LA, Kopaniak MM, Johnston RB, Jr., Henson PM. (1985). Modulation of multiple neutrophil functions by preparative methods or trace concentrations of bacterial lipopolysaccharide. Am J Pathol. 119:101.

11. Brown RD, Ambler SK, Li M, Sullivan TM, Henry LN, Crossno JT, Jr., et al. (2013). MAP kinase kinase kinase-2 (MEKK2) regulates hypertrophic remodeling of the right ventricle in hypoxia-induced pulmonary hypertension. Am J Physiol Heart Circ Physiol. 304:H269. doi:10.1152/ajpheart.00158.2012

12. Pugliese SC, Kumar S, Janssen WJ, Graham BB, Frid MG, Riddle SR, et al. (2017). A Time- and Compartment-Specific Activation of Lung Macrophages in Hypoxic Pulmonary Hypertension. J Immunol. 198:4802. doi:10.4049/jimmunol.1601692

13. Chen S, Huang T, Zhou Y, Han Y, Xu M, Gu J. (2017). AfterQC: automatic filtering, trimming, error removing and quality control for fastq data. BMC Bioinformatics. 18:80. doi:10.1186/s12859-017-1469-3

14. Kersey PJ, Staines DM, Lawson D, Kulesha E, Derwent P, Humphrey JC, et al. (2012). Ensembl Genomes: an integrative resource for genome-scale data from non-vertebrate species. Nucleic Acids Res. 40:D91. doi:10.1093/nar/gkr895

15. Dobin A, Davis CA, Schlesinger F, Drenkow J, Zaleski C, Jha S, et al. (2013). STAR: ultrafast universal RNA-seq aligner. Bioinformatics. 29:15. doi:10.1093/bioinformatics/bts635

16. Liao Y, Smyth GK, Shi W. (2014). featureCounts: an efficient general purpose program for assigning sequence reads to genomic features. Bioinformatics. 30:923. doi:10.1093/bioinformatics/btt656

17. Robinson MD, McCarthy DJ, Smyth GK. (2010). edgeR: a Bioconductor package for differential expression analysis of digital gene expression data. Bioinformatics. 26:139. doi:10.1093/bioinformatics/btp616

18. Benjamini Y, Hochberg Y. (1995). Controlling the False Discovery Rate: a Practical and Powerful Approach to Multiple Testing. Royal Statistical Society. 57:289.

19. Gu Z, Eils R, Schlesner M. (2016). Complex heatmaps reveal patterns and correlations in multidimensional genomic data. Bioinformatics. 32:2847. doi:10.1093/bioinformatics/btw313

20. D'Alessandro A, Nemkov T, Kelher M, West FB, Schwindt RK, Banerjee A, et al. (2015). Routine storage of red blood cell (RBC) units in additive solution-3: a comprehensive investigation of the RBC metabolome. Transfusion. 55:1155. doi:10.1111/trf.12975

21. Nemkov T, Reisz JA, Gehrke S, Hansen KC, D'Alessandro A. (2019). High-Throughput Metabolomics: Isocratic and Gradient Mass Spectrometry-Based Methods. Methods Mol Biol. 1978:13. doi:10.1007/978-1-4939-9236-2_2

22. Chong J, Wishart DS, Xia J. (2019). Using MetaboAnalyst 4.0 for Comprehensive and Integrative Metabolomics Data Analysis. Curr Protoc Bioinformatics. 68:e86. doi:10.1002/cpbi.86

23. Choi HJ, Jhe YL, Kim J, Lim JY, Lee JE, Shin MK, et al. (2020). FoxM1-dependent and fatty acid oxidation-mediated ROS modulation is a cell-intrinsic drug resistance mechanism in cancer stem-like cells. Redox Biol. 36:101589. doi:10.1016/j.redox.2020.101589

24. Schiavone ML, Pecorelli A, Woodby B, Ferrara F, Pambianchi E, Santucci A, et al. (2020). Mechanisms involved in the unbalanced redox homeostasis in osteoblastic cellular model of Alkaptonuria. Arch Biochem Biophys. 690:108416. doi:10.1016/j.abb.2020.108416

25. Blacker TS, Mann ZF, Gale JE, Ziegler M, Bain AJ, Szabadkai G, et al. (2014). Separating NADH and NADPH fluorescence in live cells and tissues using FLIM. Nat Commun. 5:3936. doi:10.1038/ncomms4936

26. De Ruyck J, Famerée M, Wouters J, Perpète EA, Preat J, Jacquemin D. (2007). Towards the understanding of the absorption spectra of NAD(P)H/NAD(P)+ as a common indicator of dehydrogenase enzymatic activity. Chemical Physics Letters. 450:119. doi:10.1016/j.cplett.2007.10.092

27. Stringari C, Nourse JL, Flanagan LA, Gratton E. (2012). Phasor fluorescence lifetime microscopy of free and protein-bound NADH reveals neural stem cell differentiation potential. PLoS One. 7:e48014. doi:10.1371/journal.pone.0048014

28. Zhang Q, Piston DW, Goodman RH. (2002). Regulation of corepressor function by nuclear NADH. Science. 295:1895. doi:10.1126/science.1069300

29. Williamson DH, Lund P, Krebs HA. (1967). The redox state of free nicotinamide-adenine dinucleotide in the cytoplasm and mitochondria of rat liver. Biochem J. 103:514. doi:10.1042/bj1030514

**Supplementary Figure Legend**

**Supplemental Figure 1: The effects of IL6 alone, lactate alone or combination of IL6 and lactate do not match the level of PH-CM in activating mouse BMDMs.** Naïve mouse bone marrow derived macrophages (BMDMs) were treated with cytokine IL6 (2, 20ng/ml), metabolite lactate (25mM), combination of IL6+lactate, or media conditioned by pulmonary artery adventitial fibroblasts isolated from 2-week old hypoxia induced pulmonary hypertensive calves (PH-CM) or left untreated (UNX). qPCR analysis showed fold change of *Arg1, Il1b, Vegf, Ass1*, and *Asl* expression relative to untreated BMDMs. **p*≤0.05,** *p*≤0.01,*** *p*≤0.001,**** *p*≤0.0001, significantly different compared to UNX; ^^^^ *p*≤0.0001, significantly different compared to 2ng/ml IL6; #### *p*≤0.0001, significantly different compared to lactate.

**Supplemental Figure 2: Flow chart for RNA-seq data analysis.**

Naïve bone marrow derived macrophages (BMDMs) were treated with media conditioned by pulmonary artery adventitial fibroblasts isolated from 2-weeks old hypoxia induced pulmonary hypertensive (PH-CM) or age-matched control calves (CO-CM), or left untreated (UNX). Of 53,465 genes sequenced, 12,191 met quality control standards and with count greater than 1. 3,348 genes were further removed because neither CO-CM nor PH-CM significantly regulated the expression of these genes compared to untreated BMDMs (Cutoff criteria for differential gene expression: fold change ≥ |1.2|, and FDR ≤ 0.05). The rest of differentially expressed genes were shown in VENN diagrams. Both CO-CM and PH-CM treatment significantly altered expression of a shared set of 5,924 genes. Among these genes, there are 5,756 genes were regulated to the same direction (either up- or down-regulated) by both CO-CM and PH-CM. The rest of 168 genes were regulated to the opposite directions by CO-CM or PH-CM. There are 1,061 genes were significantly regulated by CO-CM only, 1,858 genes were significantly regulated by PH-CM only. The uniquely regulated genes by CO-CM (1,061+168=1,229) and PH-CM (1,858+168=2,026) were used for pathway analysis.

**Supplemental Figure 3. PH-CM stimulated expression of both M1 and M2 markers in macrophages.**

M1 markers are listed and encompassed by a peach oval. M2 markers are listed and encompassed by panel blue oval. Markers induced by PH-CM in BMDM are represented by central oval in the common area and highlighted in red.

**Supplemental Figure 4. Different reference genes validate qPCR results.**

Naïve bone marrow derived macrophages (BMDMs) were treated with media conditioned by pulmonary artery adventitital fibroblasts isolated from 2-weeks old hypoxia induced pulmonary hypertensive (PH-CM) or age-matched control calves (CO-CM), or left untreated (UNX). mRNA were collected and gene expression was examined by qPCR analysis. In the top panel, target gene expression is normalized to Hprt. In the bottom panel, second reference gene, 18s, was used. Although absolut fold changes vary, the induction trends are unchanged. Data is represented by fold change relative to untreated macrophages isolated from wildtype mice, and displayed as mean ± sem. * *p*<0.05, ** *p*<0.01, *** *p*<0.001, **** *p*<0.0001, compared to untreated BMDMs.

**Supplemental Figure 5.** **Genetic Interruption of HIf1a in mouse BMDMs results in attenuated activation by PH-CM.**

Mouse bone marrow derived macrophages (BMDM) wer isolated from either wildtype (WT) or *Hif1α*^fl/fl^;LysMcre (HIF1-/-) C57bl6/J mice and were treated with PH-fibroblast conditioned media (PH-CM) or left untreated (UNX). qPCR was performed to examine the macrophage activation marker (*Arg1*), inflammatory gene (*Il4ra, Socs3*), metabolic gene (*Glut1*), and vascular remodeling gene (*Vegf*). Data is shown as fold change relative to the WT untreated group and displayed as mean±se. *, *p* ≤ 0.05 compared to WT untreated; # *p* ≤ 0.05 compared to WT PH-CM treated.

**Supplemental Figure 6. Gene expression comparison of macrophages from three different species in response to bovine fibroblast conditioned media.**

Mouse BMDMs, bovine BMDMs and human monocyte derived macrophages (MDMs) were treated with bovine control and pulmonary hypertensive fibroblast conditioned media to examine whether macrophages from different species respond to bovine fibroblast conditioned media differently. mRNA were collected from untreated (UNX), CO-CM and PH-CM treated macrophages and gene expression was examined by qPCR analysis. Data is represented by fold change relative to untreated macrophages, and displayed as mean ± sem. * *p*<0.05, ** *p*<0.01, *** *p*<0.001, **** *p*<0.0001.

**Supplemental Figure 7. Control and PH-fibroblast conditioned media have distinct effects on regulating macrophage metabolism.** A) qPCR confirmed that PH-CM induced gene expression of key molecules and enzymes involved in metabolism in mouse BMDM. In order to confirm the expression of molecules and enzyme suggested by IPA in condition media treated BMDMs. mRNA were collected from untreated (UNX), CO-CM and PH-CM treated BMDMs and gene expression was examined by qPCR. Data is represented by fold change relative to untreated BMDM, and displayed as mean ± sem. * *p*<0.05, ** *p*<0.01, *** *p*<0.001, **** *p*<0.0001. B) The heatmap shows metabolites that weresignificantly regulated by PH-CM compared to untreated or CO-CM treated mouse BMDM.

**Supplemental Figure 8. The overall gene-metabolites interaction network of CO-CM or PH-CM treated BMDMs.** Differentially expressed genes and metabolites (*p* ≤ 0.05) from BMDMs treated with CO-CM or PH-CM compared to untreated BMDMs were used to identify a gene-metabolite interaction network with MetaboAnalyst (<https://www.metaboanalyst.ca/>). CO-CM treated BMDMs generated 5 small modules that do not merge into an overall integrated network. In contrast, PH-CM treated BMDMs generated a complicated gene-metabolite interaction network. Squares: metabolites, circles: genes. Colors are arbitrary and do not signify anything.

**Supplemental Figure 9. Macrophages respond to PH-CM through an integrated transcriptional and metabolic network.** Metabolites of the pathways detected in PH-CM treated vs. untreated BMDMs by mass spectrometry analysis were combined with differentially expressed genes (*p* ≤ 0.05) from RNA-seq analysis to generate gene-metabolite network pathways using Metaboanalyst Network Explorer ([www.metaboanalyst.ca](http://www.metaboanalyst.ca)). Four metabolic modules were analyzed in details: A) TCA cycle and glutamine metabolism, B) glycerophospholipids and fatty acid metabolism, C) NADH, flavin mononucleotide (FMN), D) Arginine, polyamines metabolism, and urea cycle. Genes and metabolites with established functions in macrophage phenotype are identified and their node symbols are colored based on their levels in PH-CM treated BMDMs compared to untreated BMDMs. Orange circle: increased genes. Green circle: decreased genes. Orange Square: increased metabolites. Grey Square: genes or metabolites are not significantly changed in response to PH-CM. Yellow circles: genes or metabolites not known to be significant in macrophage phenotypes. The size of the nodes is arbitrary and does not signify anything.

**Supplemental Figure 10. Macrophages exhibited metabolic reprogramming in response to PH-fibroblast conditioned media to support their functions in PH.** A schematic diagram representing the metabolic reprogramming and functions of macrophages in response to PH-CM. PH-CM treated BMDMs exhibited: 1) increased glycolysis with increased lactate production, a pro-inflammatory signal for the surrounding cells, 2) accumulation of amino acids and nucleotides, substrates for energetic and synthetic cellular programs, 3) increased pentose phosphate pathway, integral to the anti-oxidant system and a source of substrates for nucleotide synthesis, 4) increased levels of glutamine, glutamate, and α-ketoglutarate, suggesting a re-wiring of the fuel preference for the TCA cycle to promote dependence on more amino acid based sources (e.g. glutamine) rather than glucose derived carbons, which are diverted to lactate, 5) increased hydroxyglutarate, a prolylhydroxylase domain (PHD) inhibitor supportive of hypoxia inducible factor (HIF) signaling pathway, 6) accumulation of amino-sugars, gamma-glutamyls and fatty acids; 7) increased arginase and conversion of arginine to ornithine for polyamine synthesis supportive of cell growth and extra cellular matrix remodeling.
