# Supplementary figures and images for: Microenvironmental Regulation of Macrophage Transcriptomic and Metabolomic Profiles in Pulmonary Hypertension
Source: Front Immunol. 2021 Mar 31;12:640718. doi: 10.3389/fimmu.2021.640718 (PMC8044406; doi:10.3389/fimmu.2021.640718)

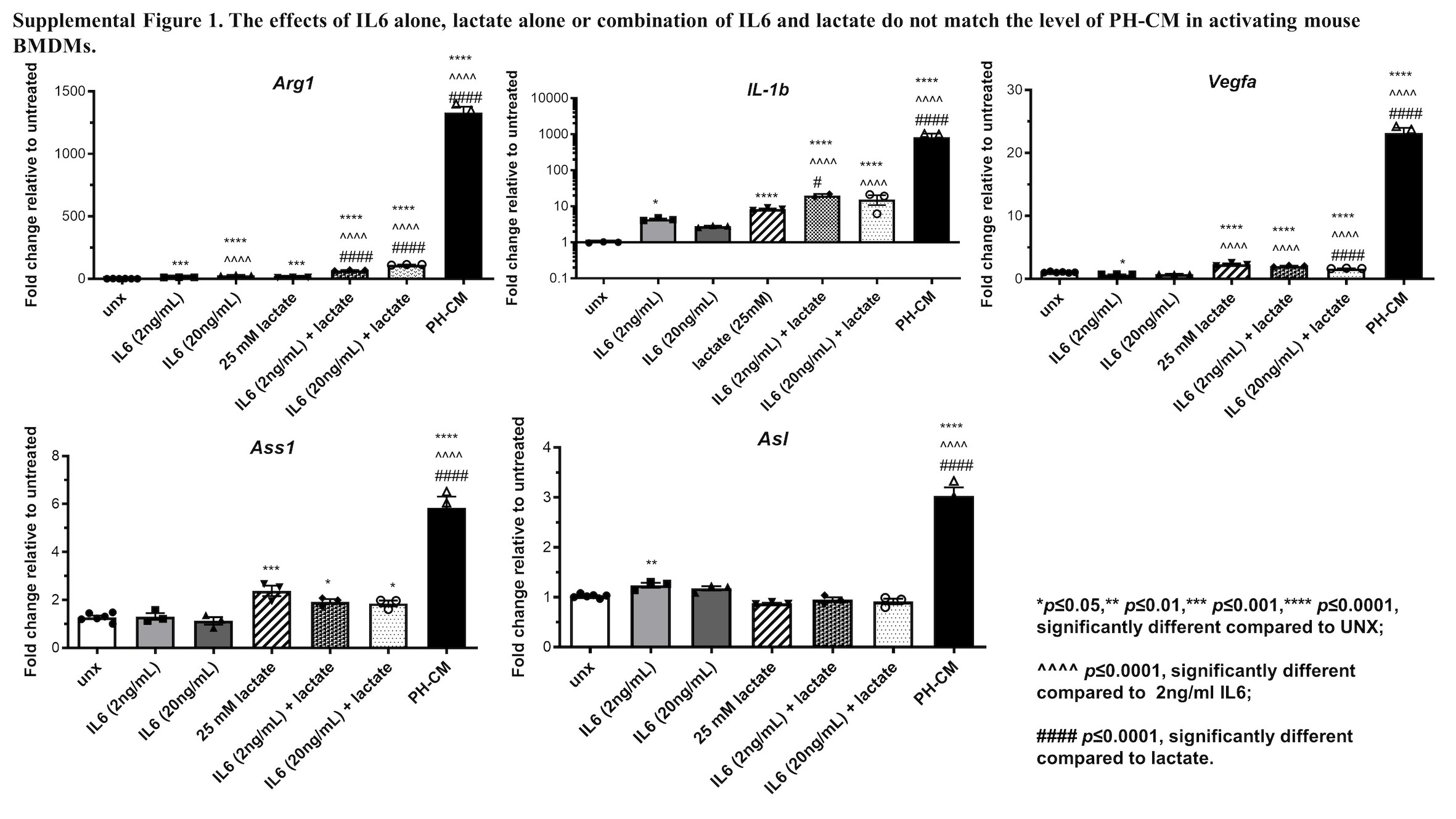

Supplement: Supplementary file 2 [file Image_1.jpg]

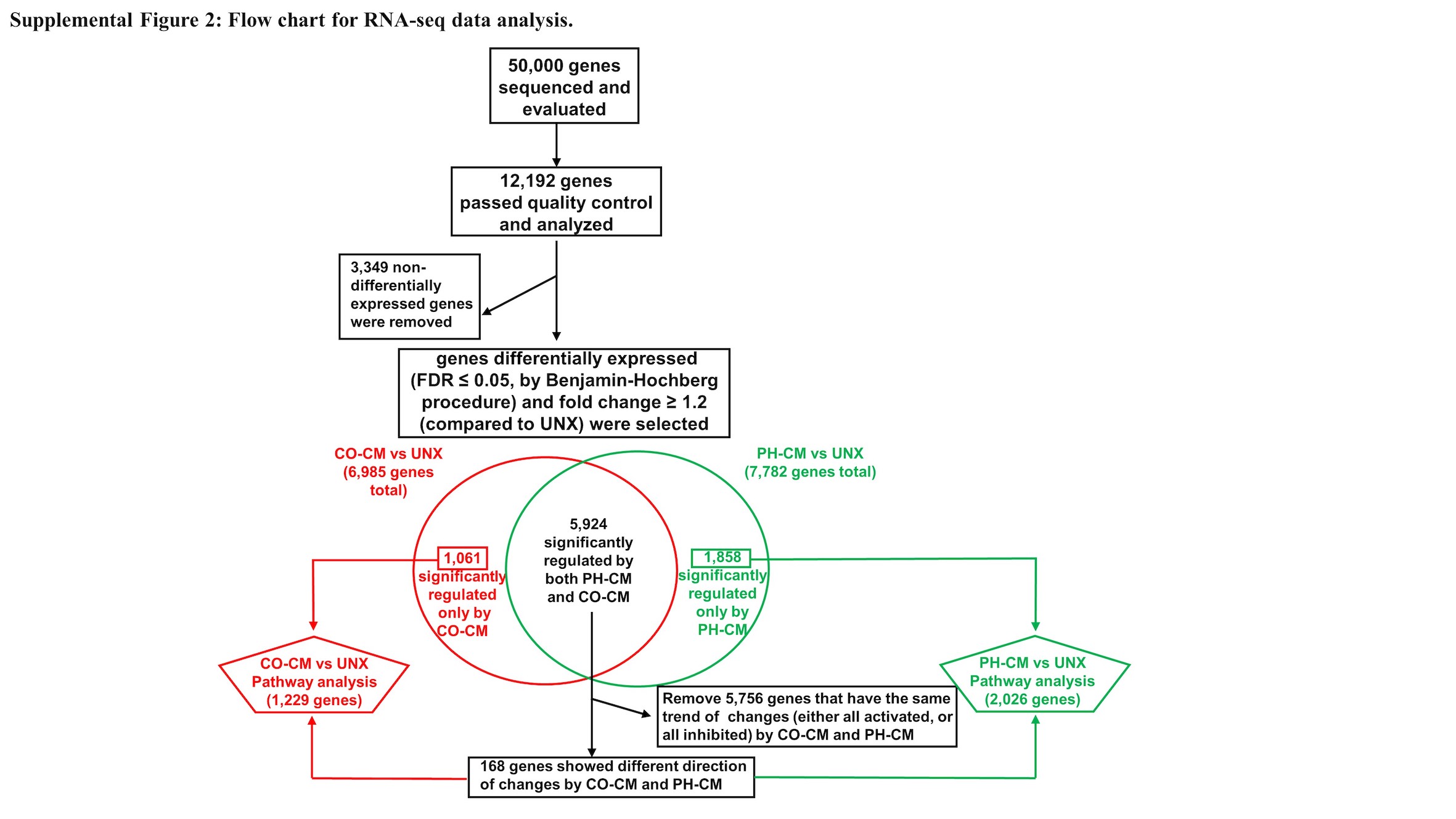

Supplement: Supplementary file 3 [file Image_2.jpg]

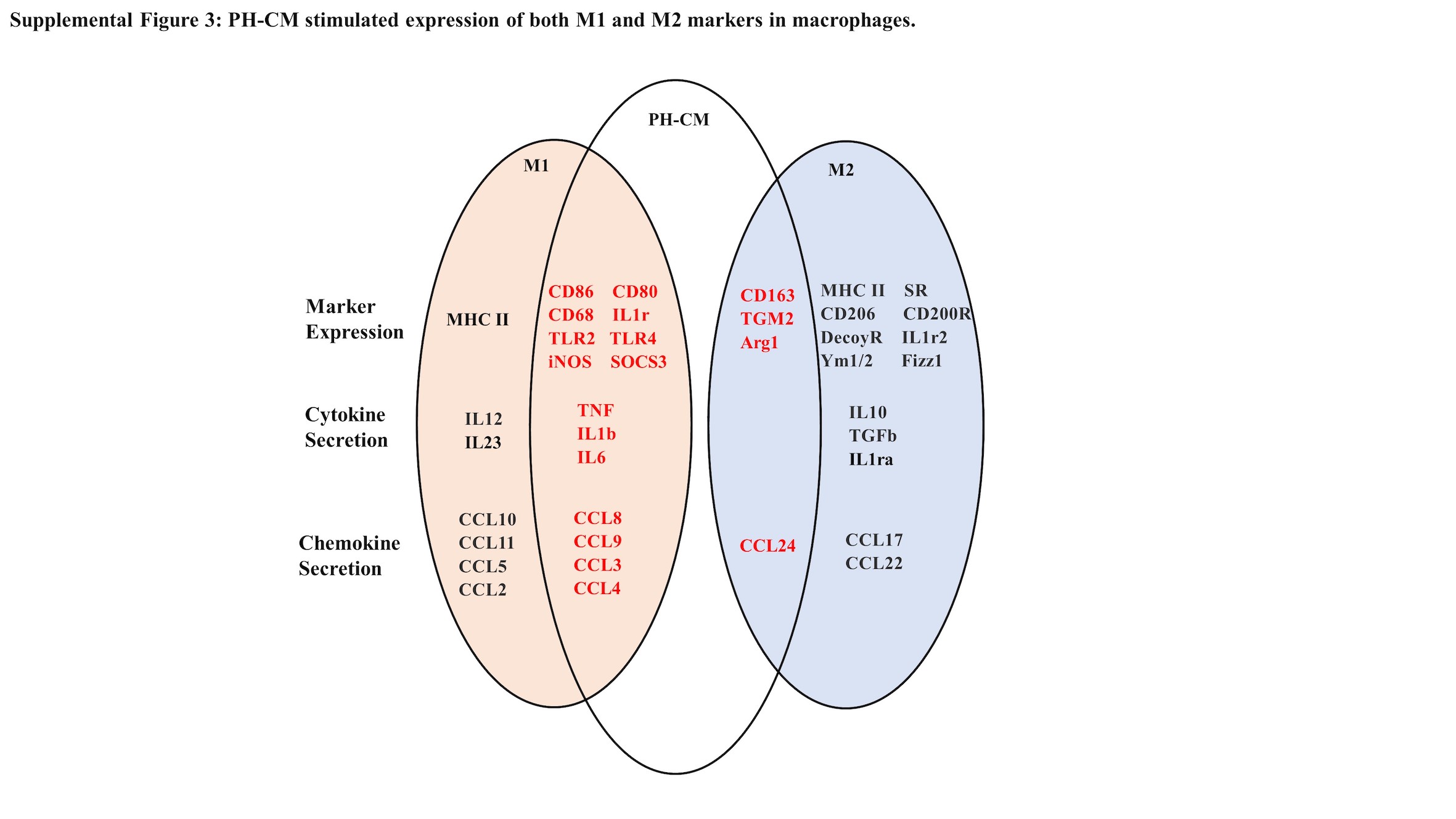

Supplement: Supplementary file 4 [file Image_3.jpg]

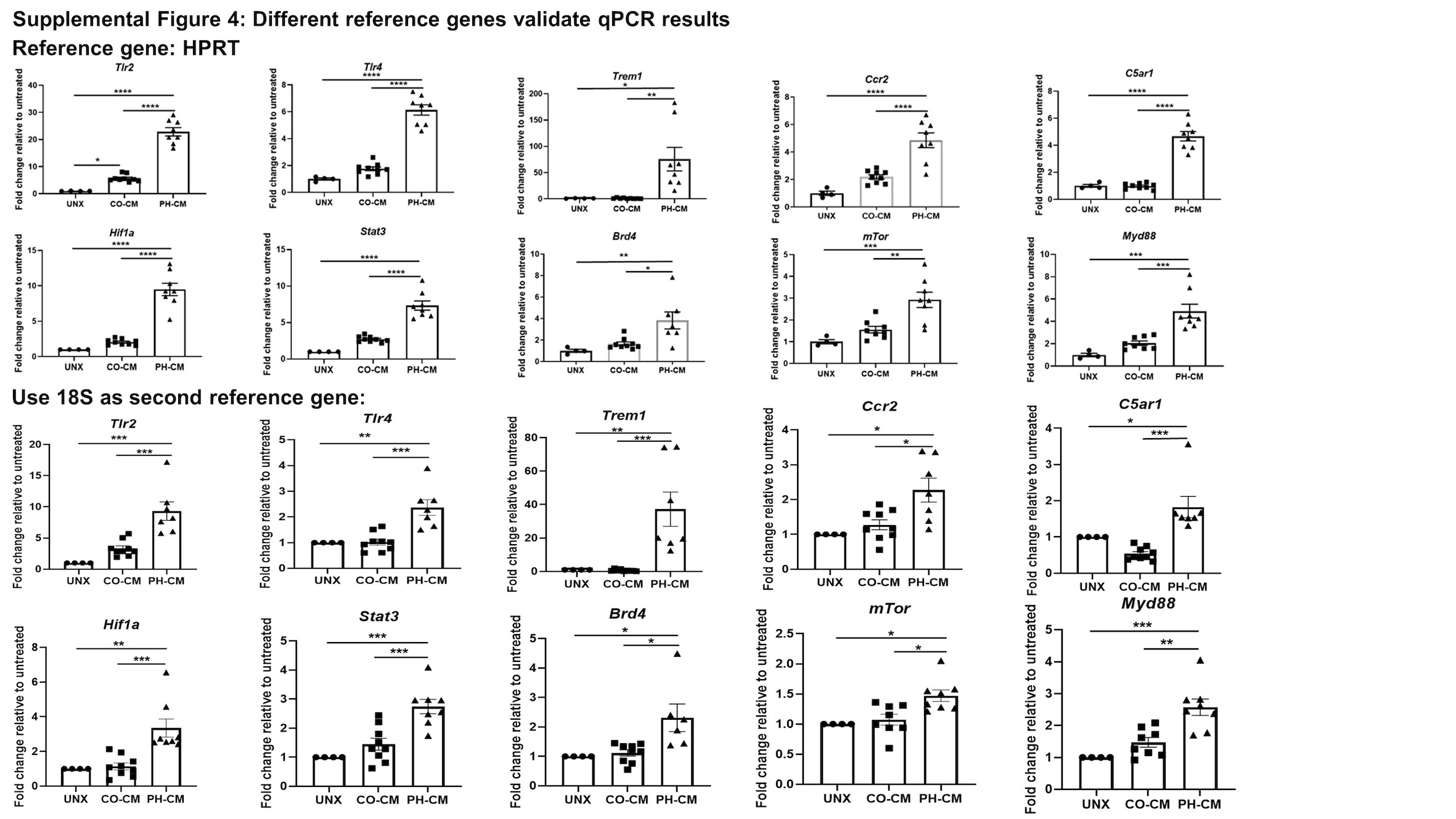

Supplement: Supplementary file 5 [file Image_4.jpg]

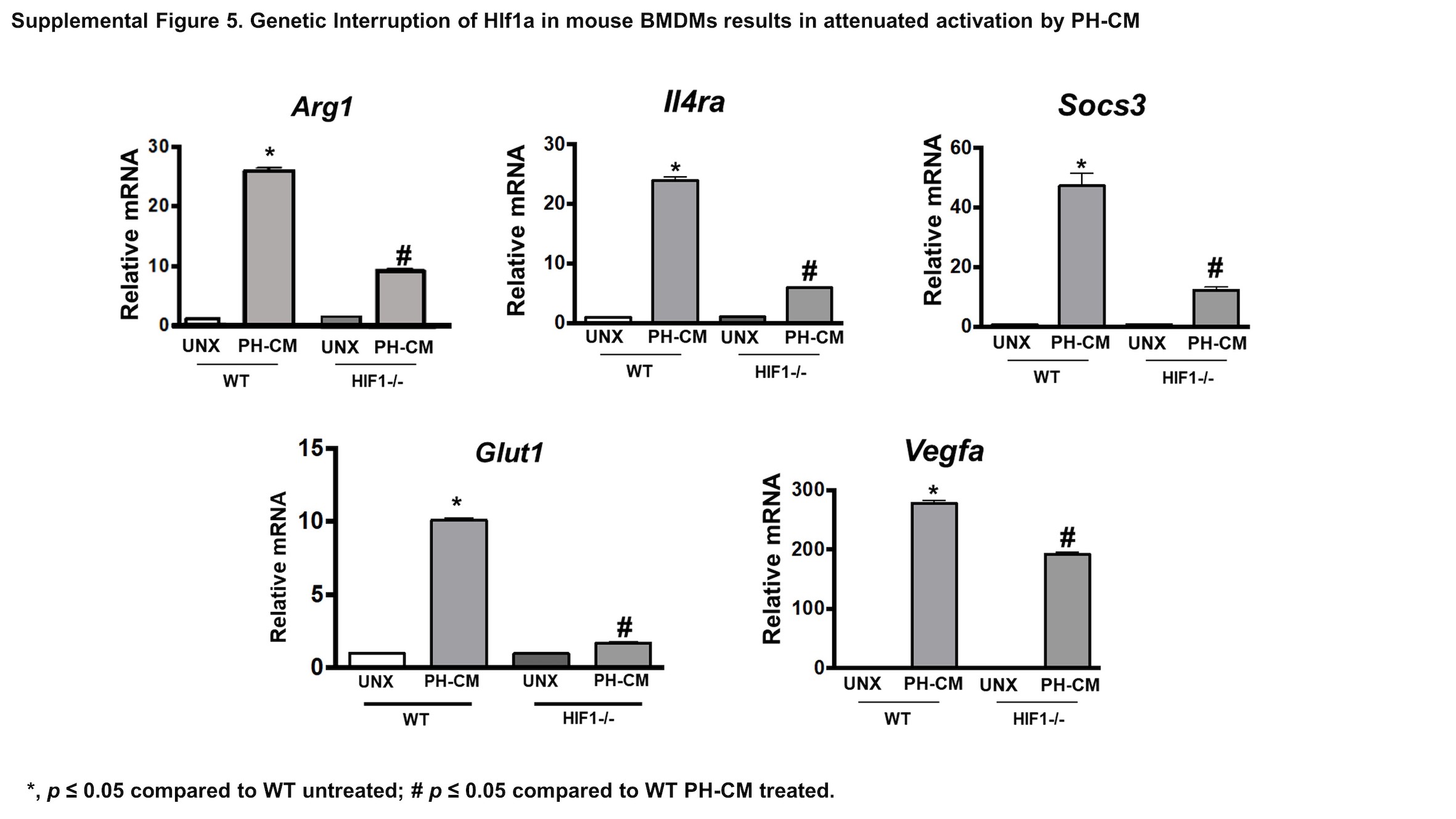

Supplement: Supplementary file 6 [file Image_5.jpg]

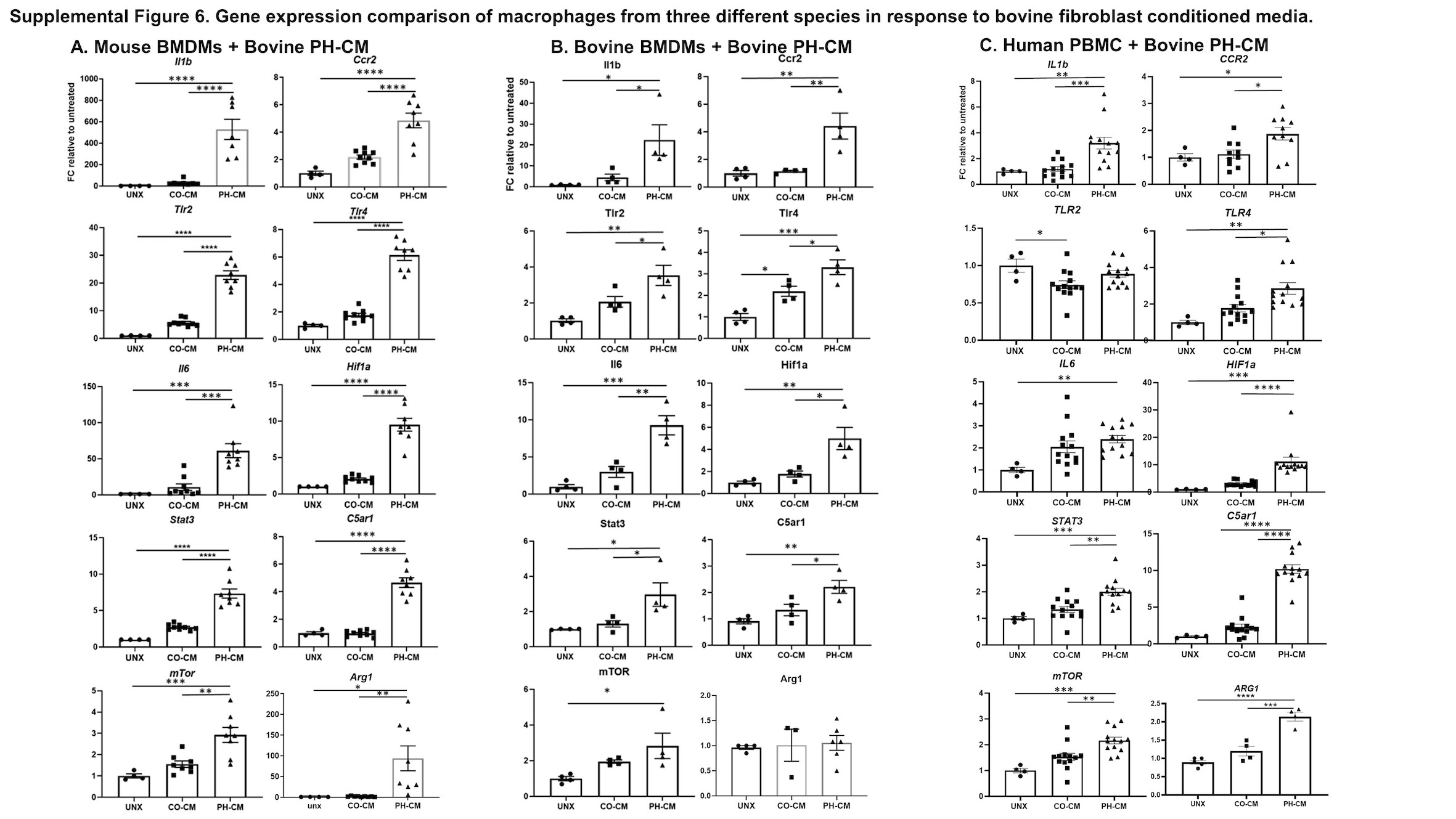

Supplement: Supplementary file 7 [file Image_6.jpg]

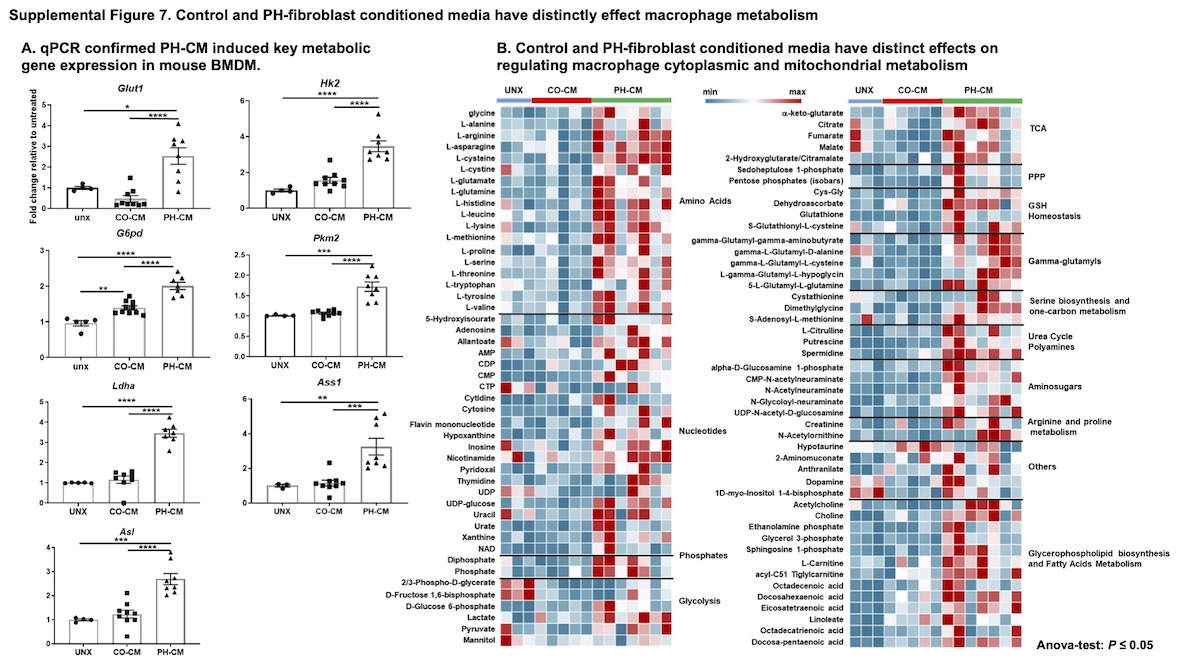

Supplement: Supplementary file 8 [file Image_7.jpg]

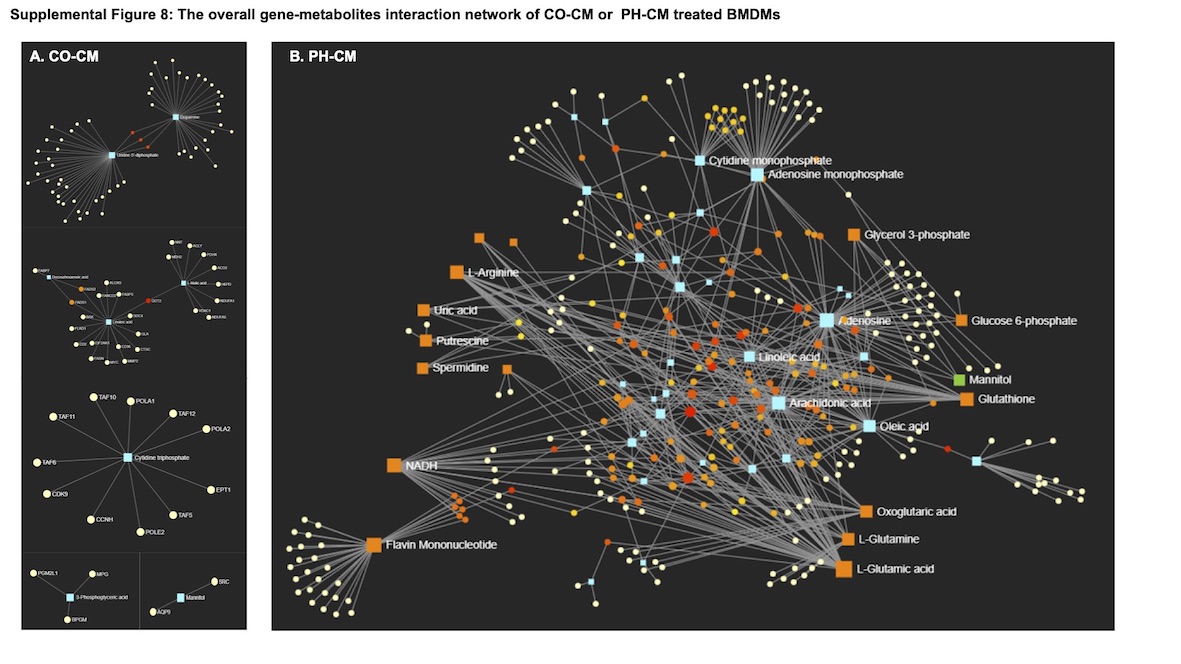

Supplement: Supplementary file 9 [file Image_8.jpg]

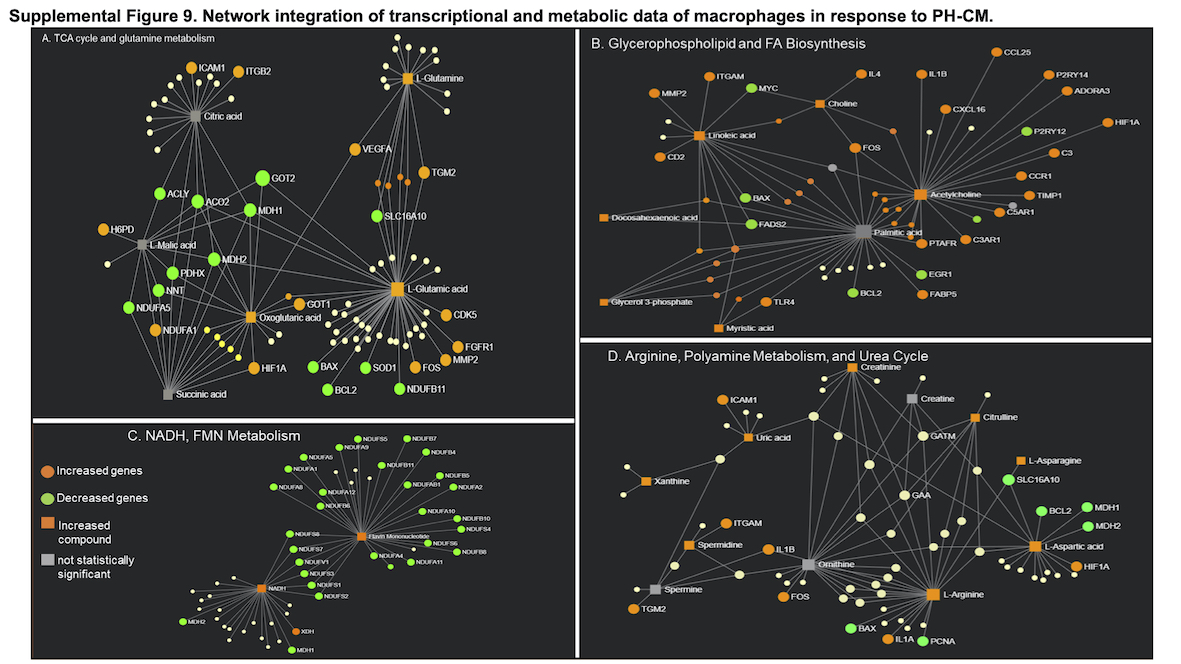

Supplement: Supplementary file 10 [file Image_9.jpg]

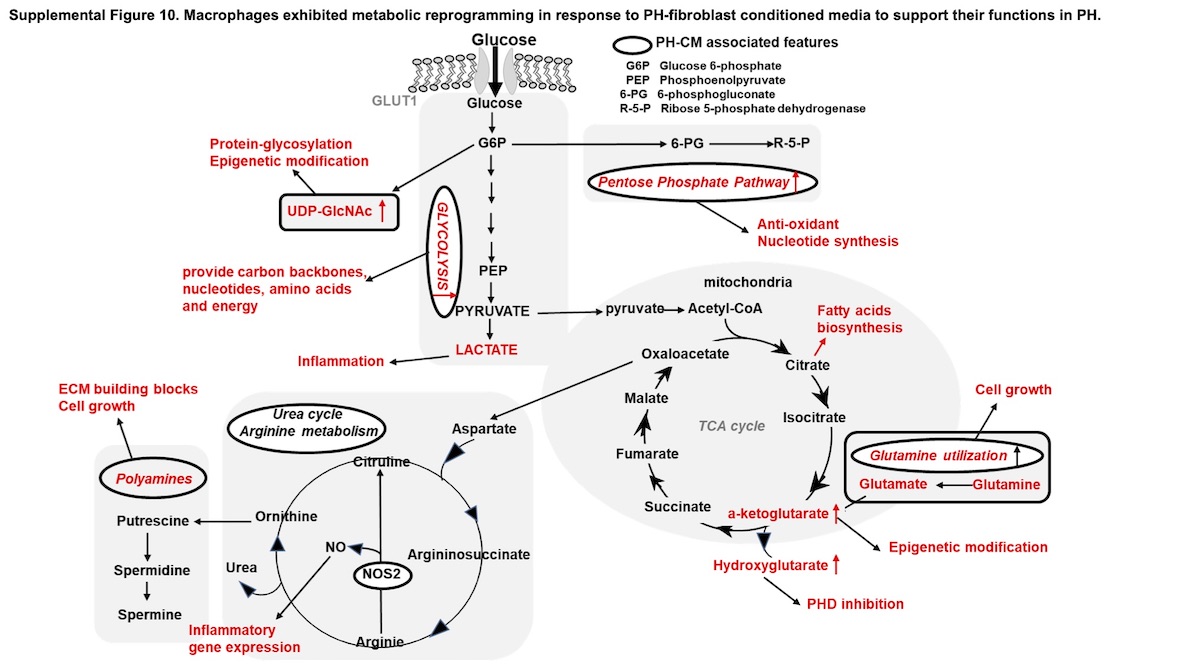

Supplement: Supplementary file 11 [file Image_10.jpg]
