# Supplementary material for: Microenvironmental Regulation of Macrophage Transcriptomic and Metabolomic Profiles in Pulmonary Hypertension
Source: Front Immunol. 2021 Mar 31;12:640718. doi: 10.3389/fimmu.2021.640718 (PMC8044406; doi:10.3389/fimmu.2021.640718)
Supplement: Supplementary file 12 [file Table_1.docx]

| Supplemental Table 1: mouse, bovine and human primers and mouse TaqMan probes | | | | | | | | |
| --- | --- | --- | --- | --- | --- | --- | --- | --- |
| Mouse real-time PCR primer sequence 5’-3’ | | | | | | | | |
| HPRT | FWD | TGGGCTTACCTCACTGCTTT | | BRD4 | FWD | AGACTCCTTCACCGGTACCCC | | |
|  | REV | CTAATCACGACGCTGGGACT | |  | REV | TGCACAGGCTGTGGAACTGG | | |
| PKM2 | FWD | ATTACCAGCGACCCCACAGAA | | MyD88 | FWD | CACCTGTGTCTGGTCCATTG | | |
|  | REV | ACGGCATCCTTACACAGCACA | |  | REV | AGGCTGAGTGCAAACTTGGT | | |
| mTOR | FWD | AAACACTTCGGAGAGCTGGA | |  |  |  |  |  |
|  | REV | CGGGTCTTCCTTGTTTGTGT | |  |  |  |  |  |
| Mouse TaqMan Probes | | | | | | | | |
| S100A4 | Mm00803372_g1 | | Slc2a1 | Mm00441480_m1 | | G6PD | Mm00658204_s1 | |
| ARG1 | Mm00475988_m1 | | CCR2 | Mm99999051_m1 | | ODC1 | Mm02019269_g1 | |
| LDHA | Mm01612132_g1 | | C5aR1 | Mm00500292_s1 | | ASS1 | Mm00711256_m1 | |
| IL1b | Mm00434228_m1 | | STAT3 | Mm01219775_m1 | | ASL | Mm01197741_m1 | |
| TLR2 | Mm00442346_m1 | | HIF1α | Mm00468869_m1 | | TREM1 | Mm01278455_m1 | |
| TLR4 | Mm00445273_m1 | | HK2 | Mn00443385_m1 | | ARG2 | Mm00477592_m1 | |
| IL6 | Mm00446190_m1 | | TNC | Mm00495662_m1 | | CTSS | Mm01255859_m1 | |
| HPRT | Mm01545399_m1 | |  |  |  |  |  |  |
| Bovine real-time PCR primer sequence 5’-3’ | | | | | | | | |
| IL1b | FWD | GAATGGAAACCCTCTCTCCC | IL6 | FWD | GTGAAAGCAGCAAGGAGACA | HIF1α | FWD | CCACCTCTGGACGTGCCTTT |
|  | REV | GCTGCAGCTACATTCTTCCC |  | REV | ATCCGTCCTTTTCCTCCATT |  | REV | TTTCTTGTCGTTCGCGCCCC |
| TLR2 | FWD | TTCTGAATGCCACAGGGCGG | STAT3 | FWD | CCTCTCAGACCCAGAAGCAC | mTOR | FWD | GCTGAAGGACTCCTCGTCAC |
|  | REV | TGCAGCCACGCCCACATCAT |  | REV | CCTGTCAACCCGTTTGTCTT |  | REV | CTCAGACCAGCAGGACACAA |
| TLR4 | FWD | ATGCCAGGATGATGGCGCGT | C5ar1 | FWD | CTGCTGACCATACCGTCCTT | CCR2 | FWD | TTGTTGGGGAGAAGTTCAGG |
|  | REV | ACCTGTACGCAAGGGTCCCA |  | REV | GACGACACACATCGTCTTGG |  | REV | CGATCTCCTGTCTCCCCATA |
| HPRT | FWD | CTGGCTCGAGATGTGATGAA | ARG1 | FWD | TGGCGATCGGCAGCATCTCT |  |  |  |
|  | REV | CAACAGGTCGGCAAAGAACT |  | REV | TCCGTGTGAGCATCCACCCA |  |  |  |
| Human real-time PCR primer sequence 5’-3’ | | | | | | | | |
| IL1b | FWD | TGGCGGCATCCAGCTACGAA | STAT3 | FWD | GGAAGAATCCAACAACGGCA | HIF1α | FWD | TTTTACCATGCCCCAGATTCA |
|  | REV | TGGCCACAACAACTGACGCG |  | REV | CAGTCACAATCAGGGAAGCA |  | REV | AGTGCTTCCATCGGAAGGACT |
| TLR2 | FWD | GATGCCTACTGGGTGGAGAA | IL6 | FWD | ACAAGCGCCTTCGGTCCAGT | mTOR | FWD | TGTCCTGCTGGTCTGAACTG |
|  | REV | CCACTTGCCAGGAATGAAGT |  | REV | TGTGTGGGGCGGCTACATCT |  | REV | TTCAGCGATGTCTTGTGAGG |
| TLR4 | FWD | GAGCACTTGGACCTTTCCAG | C5ar1 | FWD | CCCAGGAGACCAGAACATGG | CCR2 | FWD | TCAACTGGACCAAGCCACGC |
|  | REV | TCATAGGGTTCAGGGACAGG |  | REV | AGGATGTCTGGAACACGCAG |  | REV | GGGGCAATCCTACAGCCAAG |
| HPRT | FWD | ACGTCTTGCTCGAGATGTGA | ARG1 | FWD | ATGGGGACCTGCCCTTTGCT |  |  |  |
|  | REV | AATCCAGCAGGTCAGCAAAG |  | REV | TTCTGCCACCTTGCCAGCCA |  |  |  |
