# Supplementary material for: Microenvironmental Regulation of Macrophage Transcriptomic and Metabolomic Profiles in Pulmonary Hypertension
Source: Front Immunol. 2021 Mar 31;12:640718. doi: 10.3389/fimmu.2021.640718 (PMC8044406; doi:10.3389/fimmu.2021.640718)
Supplement: Supplementary file 13 [file Table_2.docx]

| **Supplemental Table 2. Information of normal donor and Human patient use for pulmonary artery specimens.** | | | | |
| --- | --- | --- | --- | --- |
| **Patient Diagnosis** | **Age** | **Gender** | **Hemodynamics** | |
|  |  |  | **mPAP** | **PVR** |
| **CO** | **47** | **ND** | **ND** | **ND** |
| **CO** | **64** | **Male** | **ND** | **ND** |
| **CO** | **63** | **Male** | **ND** | **ND** |
| **Scleroderma PAH** | **49.7** | **Female** | **45** | **ND** |
| **PH** | **30** | **Female** | **66** | **ND** |
| **Scleroderma/MCTD PAH** | **60** | **Female** | **51** | **ND** |
| **SSCPH** | **59** | **Female** | **41** | **ND** |
| **IPAH** | **52** | **Female** | **ND** | **ND** |
