# Supplementary material for: Microenvironmental Regulation of Macrophage Transcriptomic and Metabolomic Profiles in Pulmonary Hypertension
Source: Front Immunol. 2021 Mar 31;12:640718. doi: 10.3389/fimmu.2021.640718 (PMC8044406; doi:10.3389/fimmu.2021.640718)
Supplement: Supplementary file 14 [file Table_3.docx]

| Supplemental Table 3. Canonical pathways significantly regulated CO-CM and PH-CM in BMDMs. | | | |
| --- | --- | --- | --- |
| Ingenuity Canonical Pathways (CO-CM vs UNX) | -log(p-value) | z-score | n of genes |
| T Cell Exhaustion Signaling Pathway | 3.44 | -2.84 | 20 |
| Superpathway of Inositol Phosphate Compounds | 3.97 | -2.711 | 23 |
| D-myo-inositol-5-phosphate Metabolism | 4.12 | -2.683 | 20 |
| 3-phosphoinositide Biosynthesis | 4.74 | -2.558 | 22 |
| Role of Pattern Recognition Receptors in Recognition of Bacteria and Viruses | 4.2 | -2.53 | 20 |
| D-myo-inositol (1,4,5,6)-Tetrakisphosphate Biosynthesis | 4.24 | -2.524 | 19 |
| D-myo-inositol (3,4,5,6)-tetrakisphosphate Biosynthesis | 4.24 | -2.524 | 19 |
| 3-phosphoinositide Degradation | 4.65 | -2.4 | 21 |
| Glioblastoma Multiforme Signaling | 2.52 | -2.138 | 17 |
| Glioma Invasiveness Signaling | 2.51 | -2.121 | 10 |
| PD-1, PD-L1 cancer immunotherapy pathway | 1.84 | -2.111 | 11 |
| Glycolysis I | 1.44 | -2 | 4 |
| Thrombin Signaling | 2.87 | -2 | 21 |
| Tec Kinase Signaling | 4.78 | -2 | 22 |
| Ingenuity Canonical Pathways (PH-CM vs UNX) | -log(p-value) | z-score | n of genes |
| Role of JAK1, JAK2 and TYK2 in Interferon Signaling | 1.42 | 2 | 5 |
| Granzyme B Signaling | 1.47 | 2 | 4 |
| Th1 Pathway | 1.48 | 3.051 | 16 |
| TNFR2 Signaling | 1.54 | 2.236 | 6 |
| Apelin Cardiomyocyte Signaling Pathway | 1.6 | 2.309 | 14 |
| Glutaryl-CoA Degradation | 1.68 | -2 | 4 |
| TNFR1 Signaling | 1.79 | 2.121 | 9 |
| Production of Nitric Oxide and Reactive Oxygen Species in Macrophages | 1.82 | 2.4 | 24 |
| T Cell Exhaustion Signaling Pathway | 1.91 | 2.668 | 23 |
| Eicosanoid Signaling | 1.93 | 2 | 11 |
| April Mediated Signaling | 1.97 | 2.121 | 8 |
| ILK Signaling | 2.01 | 2.683 | 25 |
| Notch Signaling | 2.04 | 2.236 | 8 |
| Sirtuin Signaling Pathway | 2.22 | 2.191 | 11 |
| Pancreatic Adenocarcinoma Signaling | 2.24 | 2.138 | 17 |
| Neuroinflammation Signaling Pathway | 2.26 | 2.874 | 11 |
| CD40 Signaling | 2.78 | 2.309 | 13 |
| Dendritic Cell Maturation | 2.85 | 3.922 | 27 |
| Type I Diabetes Mellitus Signaling | 2.92 | 2.887 | 19 |
| iNOS Signaling | 3.19 | 3.317 | 11 |
| Toll-like Receptor Signaling | 3.56 | 3.051 | 16 |
| NF-κB Signaling | 3.71 | 2.785 | 29 |
| Signaling by Rho Family GTPases | 3.97 | 2.335 | 37 |
| Oxidative Phosphorylation | 5.93 | -4.6 | 8 |
| EIF2 Signaling | 11.8 | -3.772 | 52 |
