# Supplementary material for: Microenvironmental Regulation of Macrophage Transcriptomic and Metabolomic Profiles in Pulmonary Hypertension
Source: Front Immunol. 2021 Mar 31;12:640718. doi: 10.3389/fimmu.2021.640718 (PMC8044406; doi:10.3389/fimmu.2021.640718)
Supplement: Supplementary file 15 [file Table_4.docx]

| **Supplemental Table 4: z-scores and p-values for the upstream regulators predicted by IPA in CO-CM and PH-CM treated BMDMs compared to untreated BMDMs.** | | | | | |
| --- | --- | --- | --- | --- | --- |
| **Upstream Regulators**  **(CO-CM vs UNX)** | **z-score** | **p-value** | **Upstream Regulators**  **(CO-CM vs UNX)** | **z-score** | **p-value** |
| **CCR2** | **-3.15** | **6.37E-02** | **MYC** | **-3.56** | **2.03E-04** |
| **TLR7** | **-2.95** | **3.85E-03** | **RELA** | **-2.48** | **9.50E-03** |
| **RPTOR** | **-2.61** | **2.12E-01** | **STAT1** | **-2.26** | **4.06E-03** |
| **MYD88** | **-2.45** | **3.02E-01** | **STAT3** | **-2.00** | **1.05E-03** |
|  | | | | | |
| **Upstream Regulators**  **(PH-CM vs UNX)** | **z-score** | **p-value** | **Upstream Regulators**  **(PH-CM vs UNX)** | **z-score** | **p-value** |
| **TLR2** | **2.94** | **3.00E-02** | **TP53** | **3.19** | **4.08E-15** |
| **TLR4** | **3.05** | **4.23E-06** | **RELA** | **2.82** | **1.04E-03** |
| **TREM1** | **2.04** | **8.86E-04** | **BRD4** | **2.53** | **1.28E-03** |
| **CD40** | **2.25** | **4.50E-02** | **NF-kB** | **4.46** | **2.50E-05** |
| **CCR2** | **2.03** | **5.19E-03** | **CEBRD** | **2.00** | **1.44E-02** |
| **C5aR1** | **2.07** | **2.70E-02** | **STAT4** | **3.15** | **2.52E-09** |
| **RICTOR** | **7.36** | **6.12E-21** | **HIF1a** | **2.75** | **4.40E-06** |
| **MYD88** | **2.15** | **2.05E-04** | **YAP1** | **2.27** | **4.00E-03** |
|  |  |  | **STAT3** | **2.03** | **5.44E-04** |
