# Supplementary material for: Microenvironmental Regulation of Macrophage Transcriptomic and Metabolomic Profiles in Pulmonary Hypertension
Source: Front Immunol. 2021 Mar 31;12:640718. doi: 10.3389/fimmu.2021.640718 (PMC8044406; doi:10.3389/fimmu.2021.640718)
Supplement: Supplementary file 16 [file Table_5.docx]

| **Supplemental Table 5: Comparison of gene expression levels among mouse BMDMs, bovine BMDMs and human MDM cells treated with bovine or human fibroblast CM.** | | | |
| --- | --- | --- | --- |
| **Genes**  **(PH-CM vs UNX)** | **Mouse BMDM +**  **Bovine PH-CM** | **Bovine BMDM +**  **Bovine PH-CM** | **Human MDM +**  **Bovine PH-CM** |
| **IL-1b** | **527.8 ± 90.75****** | **22.39 ± 6.11*** | **3.20 ± 0.67**** |
| **IL6** | **61.09 ± 11.80***** | **9.27 ± 1.24***** | **2.40 ± 0.44**** |
| **TLR2** | **22.88 ± 1.75****** | **3.54 ± 0.52**** | **0.89 ± 0.10** |
| **TLR4** | **6.13 ± 0.45****** | **3.31 ± 0.36***** | **2.86 ± 0.52**** |
| **STAT3** | **7.33 ± 0.70****** | **2.97 ± 0.56*** | **2.0 ± 0.24***** |
| **HIF1α** | **9.49 ± 0.97****** | **4.99 ± 0.85**** | **11.18 ± 2.17***** |
| **MYD88** | **4.92 ± 0.72***** | **2.09 ± 0.49** | **2.17 ± 0.26***** |
| **mTOR** | **2.93 ± 0.43***** | **2.83 ± 0.60*** | **2.17 ± 0.26***** |
| **CCR2** | **4.86 ± 0.61****** | **4.42 ± 0.79**** | **1.87 ± 0.34*** |
| **C5AR1** | **4.67 ± 0.39****** | **2.21 ± 0.28*** | **10.21 ± 0.95****** |
| **Arg1** | **94.09 ± 30.76 *** | **1.22 ± 0.18** | **2.14 ± 0.14****** |
| **Data is presented by fold change relative to untreated macrophages and is shown as mean ± sem.*p< 0.05, **p < 0.01, ***p<0.001, ****p < 0.0001, compared to untreated macrophages.** | | | |
